# Atp7b-dependent choroid plexus dysfunction causes transient copper deficit and metabolic changes in the developing mouse brain

**DOI:** 10.1371/journal.pgen.1010558

**Published:** 2023-01-10

**Authors:** Clorissa L. Washington-Hughes, Shubhrajit Roy, Herana Kamal Seneviratne, Senthilkumar S. Karuppagounder, Yulemni Morel, Jace W. Jones, Alex Zak, Tong Xiao, Tatiana N. Boronina, Robert N. Cole, Namandjé N. Bumpus, Christopher J. Chang, Ted M. Dawson, Svetlana Lutsenko

**Affiliations:** 1 Department of Physiology, Johns Hopkins University School of Medicine, Baltimore, Maryland; 2 Department of Medicine, Division of Clinical Pharmacology, Johns Hopkins University School of Medicine, Baltimore, Maryland; 3 Neurodegeneration and Stem Cell Program, Institute for Cell Engineering, Johns Hopkins University School of Medicine, Baltimore, Maryland; 4 Department of Neurology, Johns Hopkins University School of Medicine, Baltimore, Maryland; 5 Department of Pharmaceutical Sciences, University of Maryland School of Pharmacy, Baltimore, Maryland; 6 Department of Chemistry, University of California Berkeley, California, United States of America; 7 Department of Biological Chemistry Johns Hopkins University School of Medicine, Baltimore, Maryland; 8 Department of Molecular and Cell Biology, University of California Berkeley, California; 9 Helen Wills Neuroscience Institute, University of California Berkeley, California; 10 Solomon H. Snyder Department of Neuroscience, Johns Hopkins University School of Medicine, Baltimore, Maryland; United States of America; Children’s National Medical Center, George Washington University of the Health Sciences, UNITED STATES

## Abstract

Copper (Cu) has a multifaceted role in brain development, function, and metabolism. Two homologous Cu transporters, Atp7a (Menkes disease protein) and Atp7b (Wilson disease protein), maintain Cu homeostasis in the tissue. Atp7a mediates Cu entry into the brain and activates Cu-dependent enzymes, whereas the role of Atp7b is less clear. We show that during postnatal development Atp7b is necessary for normal morphology and function of choroid plexus (ChPl). Inactivation of Atp7b causes reorganization of ChPl’ cytoskeleton and cell-cell contacts, loss of Slc31a1 from the apical membrane, and a decrease in the length and number of microvilli and cilia. In ChPl lacking Atp7b, Atp7a is upregulated but remains intracellular, which limits Cu transport into the brain and results in significant Cu deficit, which is reversed only in older animals. Cu deficiency is associated with down-regulation of Atp7a in locus coeruleus and catecholamine imbalance, despite normal expression of dopamine-β-hydroxylase. In addition, there are notable changes in the brain lipidome, which can be attributed to inhibition of diacylglyceride-to-phosphatidylethanolamine conversion. These results identify the new role for Atp7b in developing brain and identify metabolic changes that could be exacerbated by Cu chelation therapy.

## Author summary

Copper (Cu) plays a critical role in the development and function of human brain, and Cu mis-balance is associated with numerous neuropathologies. The key transporters maintaining brain Cu homeostasis have been identified, however little is known about regulation of Cu entry into the brain. We found that the Cu transporter Atp7b is required for normal Cu entry into the brain during postanatal development in mice. Loss of Atp7b function causes morphological changes in choroid plexus, including the remodeling of its cytoskeleton, which impact essential Cu transporters Atp7a and Slc31a. This results in an apparent inability of Atp7a to transport Cu into cerebrospinal fluid, a transient Cu deficit in the brain, and associated misbalance of catecholamines and lipids. Our findings help to understand the neuropathology of human Wilson disease, which is caused by inactivating mutations in ATP7B.

## Introduction

**Fig 1:**
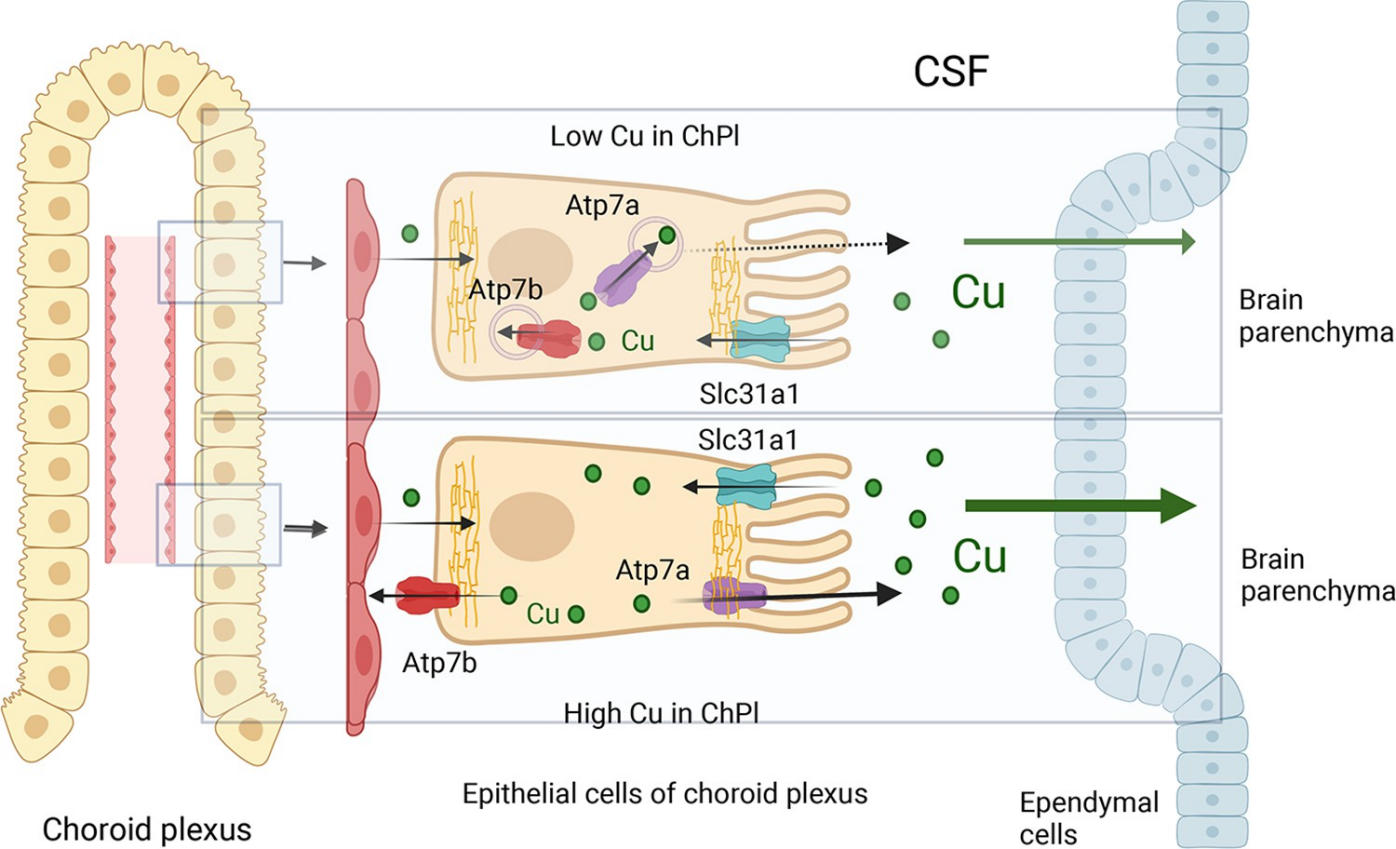
Cartoon depicting current understanding of the copper (Cu) transport in choroid plexus (ChPl). Two homologous Cu transporters, Atp7a and Atp7b regulate Cu transport in the epithelial cells of ChPl. In basal conditions and in low Cu, Atp7a and Atp7a have predominantly intracellular localization, where they sequester Cu into the lumen of the secretory pathway compartments. Dashed arrow indicates the ability of Atp7a to traffic to the plasma membrane under basal conditions and export Cu to the CSF. When Cu is elevated, Atp7a relocates towards the apical membrane and facilitates Cu transport to CSF. Atp7b traffics to the basolateral membrane to transfer excess Cu to the circulation. How these activities are coordinated is not known. Ctr1 (Slc31a1), located at the apical membrane of epithelial cells, transports Cu from the CSF into epithelial cells. Whether Cu crosses the basolateral membrane via Ctr1 or other mechanisms is unknown.

**Fig 2:**
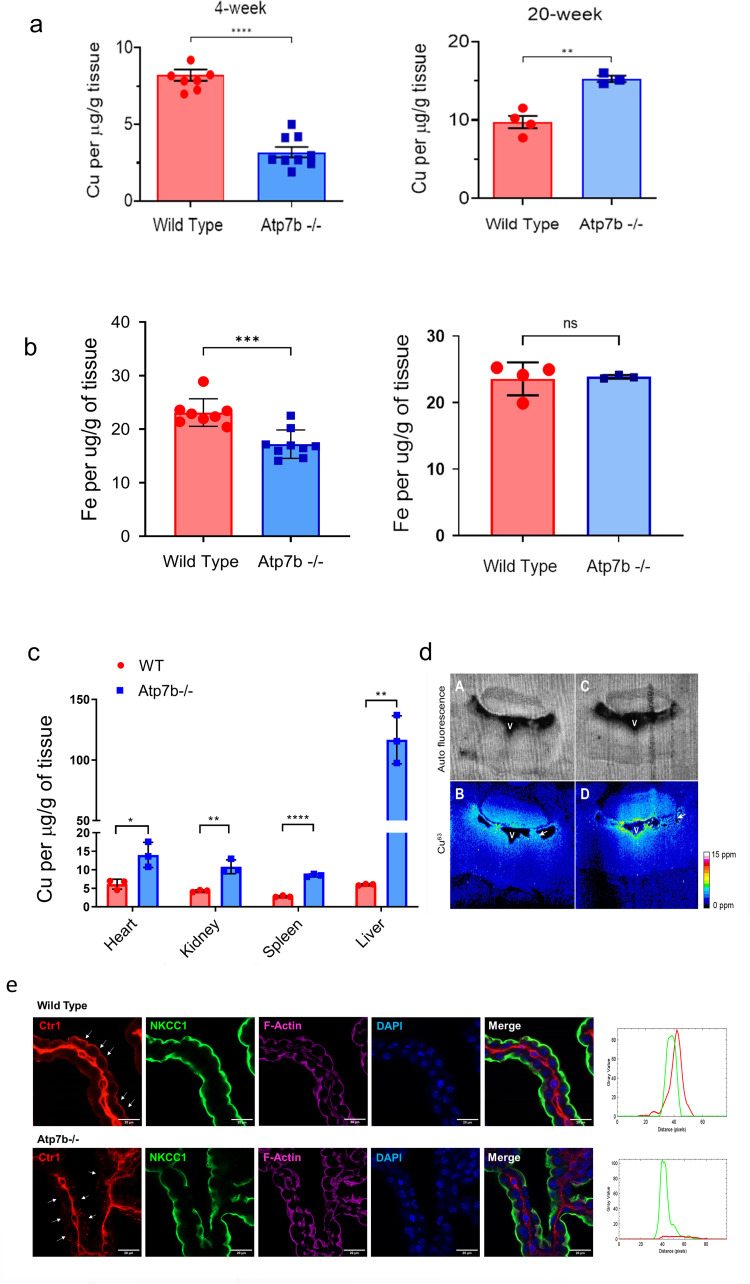
***Atp7b***^***-/-***^ **mice brain show a tissue-specific Cu deficit at 4 weeks after birth** (a) Cu levels in brains from 4 weeks old and 20 weeks old *Atp7b*^*-/-*^ mice compared to the age matched wild type controls (n = 8 for 4 week-old groups and n = 4 –for 20 week old groups). (b) The Fe levels were also reduced in 4 weeks old *Atp7b*^*-/-*^ mice compared to wild type. No significant difference in Fe levels were observed in 20 weeks old *Atp7b*^*-/-*^ and wild type animals. (c) Unlike brain, Cu levels in tissues such as heart, kidney, spleen, and liver were significantly elevated in 4 weeks old *Atp7b*^*-/-*^ mice compared to the age matched controls (n = 3 per group). The data were analyzed using unpaired two tailed t-test. p ≤ 0.05 was considered statistically significant. * p ≤ 0.05, ** p ≤ 0.01, and **** p ≤ 0.0001. (d) Maps of Cu distribution in tissue sections from control (A,B) and *Atp7b*^*-/-*^ (C,D) mice. Images shown are coronal sections of brain at approximate Bregma -5.80mm. A and C are auto fluorescence from tissue. B and D are concentration of Cu63 measured by Laser Ablation Inductively Coupled Plasma Mass Spectrometry (LA-ICP-MS). Scale bar is 200μm. Arrows point at choroid plexus. V stands for ventricle. The colorimetric scale bar represents copper concentrations ranging from 0 to 15 ppm. (e) Immunostaining of Ctr1 (red), apical membrane marker NKCC1 (green) and F-actin (magenta) in ChPl from control mice (upper panel) and *Atp7b*^*-/-*^ mice (lower panel). Apical membranes of ChPl epithelial cells are indicated with white arrow. Scale bar, 10 μm. Colocalization of Ctr1, NKCC1 and F-actin on the apical and basolateral membrane was confirmed using RGB profile plot from ImageJ software.

Copper (Cu) is a transition metal that regulates brain metabolic activities and signaling [1], and the loss of Cu balance has numerous negative consequences for brain development and function. Cu is supplied to the brain by the Cu transporter ATP7A, which exports Cu from the epithelial cells of choroid plexus (ChPl) into the cerebrospinal fluid (CSF) -**Fig 1** and [2]. Inactivation of ATP7A in patients with Menkes disease causes Cu-deficiency in the brain, numerous metabolic abnormalities (including catecholamine misbalance), delayed neurodevelopment, and death in the early childhood [3].

Wilson disease (WD) is another disorder of Cu homeostasis caused by mutations in the Cu-transporter ATP7B [4,5]. WD patients can present with tremor, ataxia, dysphagia [6–8], disrupted serotonin, dopamine (DA), norepinephrine (NE) metabolism, depression, and behavioral changes [7,9]. The molecular links between ATP7B inactivation and these pathologies remain poorly understood despite significant efforts and some progress in recent years [10–13]. Psychiatric WD can be misdiagnosed as schizophrenia [14,15]; and the delays in the diagnosis and treatment of neurologic WD are common and can be fatal [16].

These challenges are caused, in part, by very limited understanding of ATP7B role(s) in the brain. ATP7B is expressed in many brain regions, including choroid plexus [17], locus coeruleus [18], cerebellum [19], and others. The general function of ATP7B is to transport Cu from the cell cytosol into the lumen of secretory pathway for incorporation into Cu-dependent enzymes and to sequester excess Cu in vesicles for further export from cells (**Fig 1**). Specific contribution of this activity to cell metabolism depends on cell type and may vary considerably between tissues [20]. Thus, while the negative impact of ATP7B inactivation on brain metabolism is evident from WD manifestations, which specific cellular functions are disrupted by ATP7B inactivation remains unclear.

There is ample evidence showing that inactivation of ATP7B is associated with a time-dependent accumulation of Cu in tissues—first in the liver—and then elsewhere. In the animal models of WD, such as *Atp7b*^*-/-*^ mice and *tx* mice, Cu reaches its highest level in the liver at 6–8 weeks after birth [21]. In the brain, initial Cu elevation is observed at 10–16 weeks, and Cu levels continue to increase as animals age [22–24]. It is believed that this gradual Cu accumulation eventually leads to pathology development [5]. However, clinical reports suggest a more complex picture. WD patients may experience acute psychotic episodes and other behavioral changes years before other physical and neurologic changes develop; in one study, almost 50% of neurologic WD patients showed abnormal MRI before clinical manifestations [25]. These results suggest that a developing brain requires ATP7B function and that the loss of this function may disrupt brain metabolism before the onset of clinical symptoms.

Here, we tested this hypothesis in the established mouse model of WD, *Atp7b*^*-/-*^ mice. We found that during postnatal brain maturation (at 4 weeks after birth) the *Atp7b*^*-/-*^ brain had unexpected and significant Cu deficit. We provide evidence that Cu deficiency originates from dysregulated Cu entry into the brain, which in turn impacts specific metabolic pathways in brain parenchyma. We identified choroid plexus cytoskeleton as an important target of Atp7b-dependent Cu misbalance. Taken together, these results demonstrate a new role for Atp7b during postnatal brain maturation and suggest why Cu chelation during this period may have a negative outcome.

## Results

### Cu levels are low in the *Atp7b*^-/-^ mouse brain at 4 weeks after birth

Atp7b is expressed in various cell types of the central nervous system (https://www.proteinatlas.org/ENSG00000123191-ATP7B/brain); in some neurons, ATP7B protein is detected as early as at two weeks after birth [19]. Yet, the role of Atp7b in the brain, especially during brain maturation, is largely unknown. To better understand this role, we first determined how Atp7b inactivation affected the whole brain Cu content during postnatal development. Cu levels were analyzed in control and *Atp7b*^*-/-*^ brains homogenates at 4 weeks after birth (developing brain) and compared to those at 20 weeks after birth (adults). As expected, Cu was elevated in the brain of the 20-weeks *Atp7b*^*-/-*^ mice when compared to the age-matched controls (**Fig 2A**). However, at 4 weeks, Cu levels in *Atp7b*^*-/-*^ brains were significantly lower than in control animals (**Fig 2A**). This difference was not due to age-dependent changes of Cu levels in control animals, which were similar at 4 and 20 weeks. Rather, during postnatal development, *Atp7b*^*-/-*^ brain showed significant Cu deficit, which was later followed by Cu accumulation. The iron (Fe) content, which can be influenced by Cu levels, also showed decrease in *Atp7b*^*-/-*^ mice at 4 weeks compared to controls (**Fig 2B**). The decrease in Fe was less significant than Cu; and no difference in Fe levels between control and *Atp7b*^*-/-*^ brains was seen at 20 weeks (**Fig 2B**).

Cu deficit in the *Atp7b*^*-/-*^ brain could be caused by the entrapment of dietary Cu in the liver, where Atp7b normally maintains Cu homeostasis, and thus lower availability of Cu to other organs. However, analysis of the Cu content in several peripheral tissues showed Cu accumulation in all *Atp7b*^*-/-*^ samples except the brain. Thus, Cu deficit in the young brain was tissue-specific (**Fig 2C**).

Low Cu in the brain parenchyma can also be a result of enhanced Cu efflux from the ependymal cells into the CSF. However, laser-ablation-ICPMS imaging of the ependymal layer of the 4^th^ ventricle did not show lower Cu levels in the ependymal layer. The intensity of Cu signal was the same as in control cells or possibly higher, which was inconsistent with an accelerated loss of Cu from these cells (**Fig 2D**).

The levels of Cu in the CSF are also modulated by the high-affinity Cu uptake transporter Ctr1 (SLC31A1) -**Fig 1**. When Cu is elevated in CSF, Ctr1 transfers Cu from CSF into the ChPl epithelial cells for subsequent export by Atp7b [26]. Increased Ctr1 expression in ChPl may shift Cu distribution away from brain parenchyma towards ChPl; consequently, we compared the Ctr1 levels in ChPl of control and *Atp7b*^*-/-*^ mice. In control 4 weeks mice, immunostaining of Ctr1 in ChPl of the fourth ventricle shows expected signal at the apical membrane of epithelial cells (the polarity of choroid plexus is opposite to other epithelial cells [27]). This targeting was confirmed by colocalization with the apical membrane marker NKCC1 [28] (**Figs 2E and S1)** (Strong signal at the basolateral aspect of epithelial cells appears to be associated with stroma/stromal cells, S1 Fig, but further studies are needed). In *Atp7b*^-/-^ mice, the apical Ctr1 staining is lost (**Fig 2E**), arguing against enhanced Cu transfer from the brain into ChPl as a cause of brain Cu deficit.

Taken together, the results pointed to a diminished Cu entry from ChPl into the *Atp7b*^*-/-*^ brain as a likely reason for the parenchymal Cu deficiency.

### Cu transport machinery is dysregulated in choroid plexus of *Atp7b*^-/-^ mice

**Fig 3:**
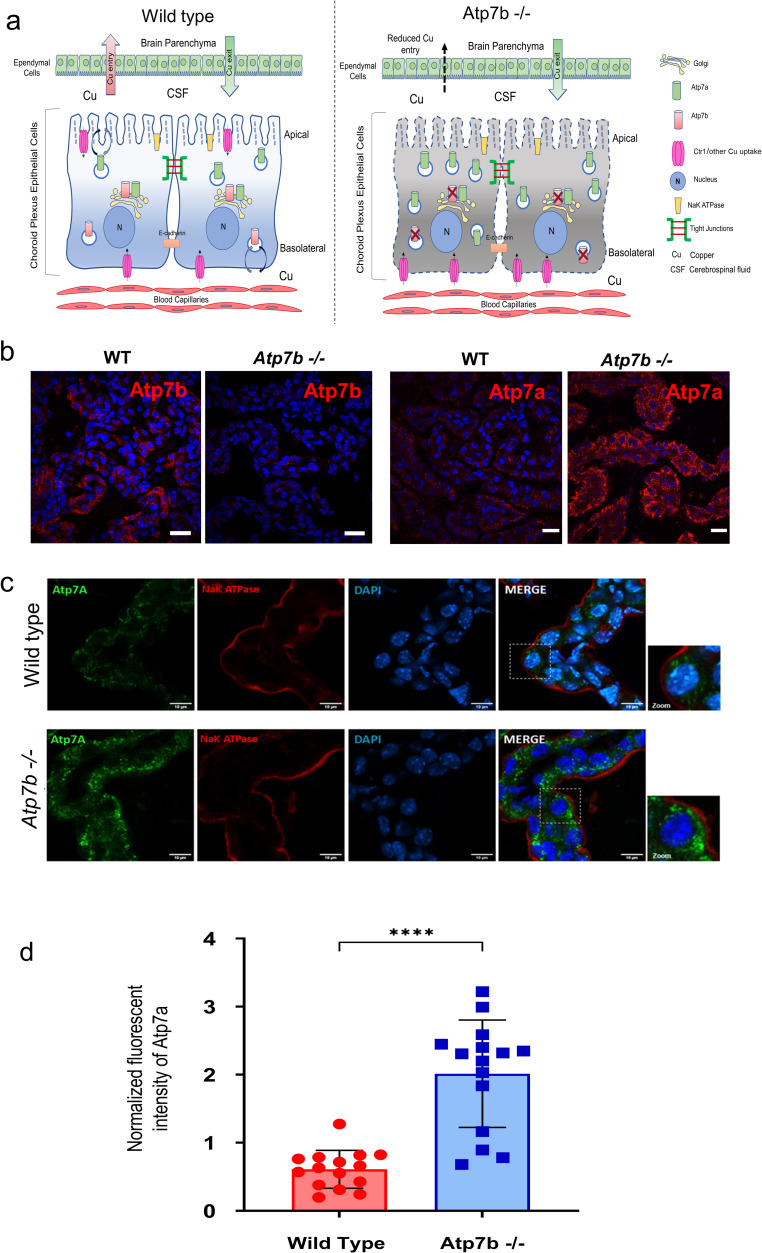
Atp7a expression and localization in control and *Atp7b*^*-/-*^ choroid plexus. (a) Schematic illustrating copper transport across the Choroid Plexus Epithelial cells (ChPE). Inactivation of Atp7b results in the increased expression of Atp7a but unaltered vesicular localization. (b) Immunofluorescence staining for Atp7b (left) and Atp7a (right) on ChPE from Atp7b -/- and wild type (+/+) mice. Scale bar, 10 μm. (c) Immunofluorescence staining for Atp7a (green) and Na,K ATPase (red) (Apical surface marker) on ChPE from control (upper panel) and *Atp7b*^*-/-*^ mice (lower panel). Scale bar, 10 μm. (n = 3 for each genotype). (d) Fluorescence intensity of immunostained Atp7a (green) was measured using color-histogram tool (ImageJ) and normalized to the fluorescent intensity of Na+/K+ ATPase (red). The data were analysed using Mann-whittney t-test in Graphpad Prism (9.4.0 software). ****P-value; <0.0001.

To test this hypothesis, we investigated the Cu transport machinery of choroid plexus. In humans, choroid plexus (ChPl) plays the major role in Cu entry into the developing brain [29]. The ChPl epithelial cells of adult animals express two ATP-driven Cu transporters, Atp7a and Atp7b [30]. Atp7a is primarily responsible for the transfer of Cu from ChPl into the CSF (**Fig 1,** [31]), whereas Atp7b is thought to return excess Cu back to the circulation [30]. We found that both transporters were expressed in normal ChPl at 4 weeks after birth (**Fig 3A and 3B**) and tested whether Atp7a down-regulation may explain diminished Cu entry into the brain. However, immunostaining showed no decrease in Atp7a expression in the *Atp7b*^*-/-*^ ChPl; in fact, the Atp7a signal was stronger when compared to the age-matched control (**Fig 3B**). Thus, Atp7a is available to facilitate Cu delivery to the brain.

For Atp7a to transport Cu to the CSF, it has to traffic to the apical membrane. Inactivation of Atp7b is expected to block Cu transport via basolateral membrane, increase cytosolic Cu and trigger trafficking of Atp7a towards the apical membrane [17]. However, in *Atp7b*^*-/-*^ ChPl the intracellular localization of Atp7a was not changed: it was mostly vesicular in either genotype (**Fig 3B**). Co-staining with the plasma membrane marker Na,K-ATPase found no significant enrichment of Atp7a at the apical membrane (**Fig 3C**). The intracellular rather than apical localization of Atp7a suggested that Atp7a trafficking may be impaired limiting Cu efflux into the CSF. We attempted to measure Cu levels in the CSF directly, but small size of animals made it difficult to get sufficient amount of CSF; in one pair of age-matched mice, which we were able to analyze, the CSF levels were lower in *Atp7b*^*-/-*^ animals.

### Atp7b-deficient ChPl has altered morphology and a decreased number of microvilli and cilia

**Fig 4:**
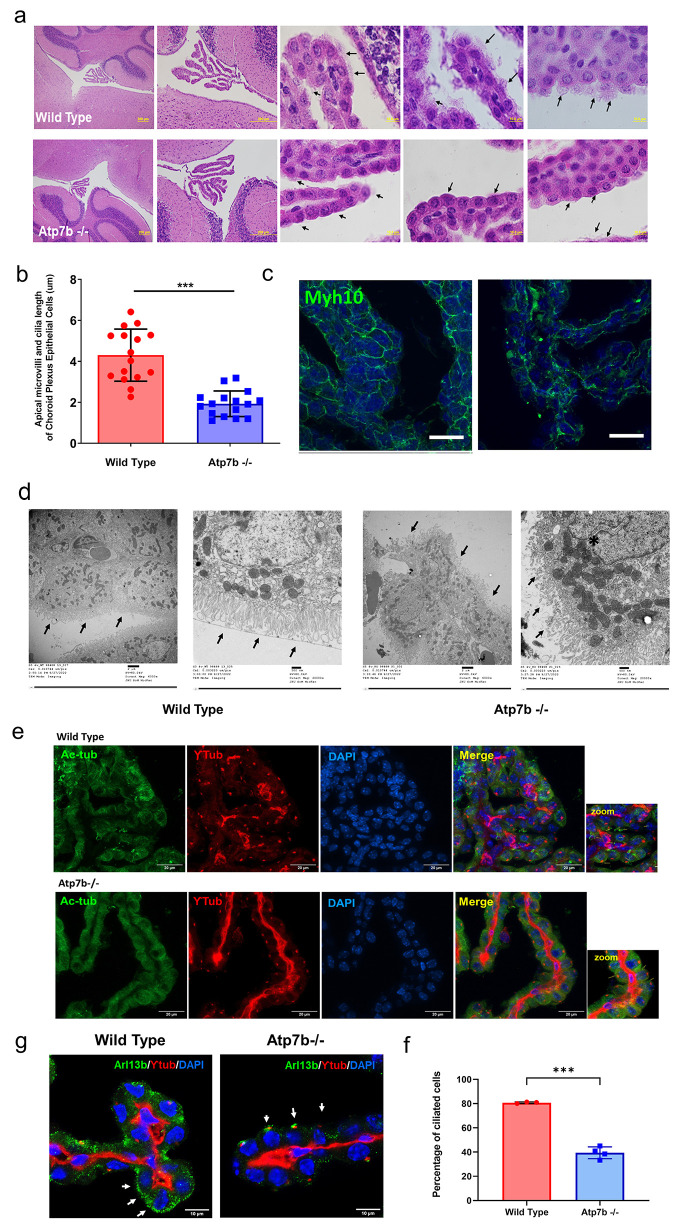
Changes in apical microvilli and ciliated cells in ChPl in *Atp7b*^*-/-*^ mice. (a) H&E staining of sagittal sections from *Atp7b-/-* and control mouse brains showing 4th ventricle choroid plexus. Scale bar; 200 μm (first two panels). Scale bar; 10 μm (last three panels). Arrow heads indicate the microvilli and ciliary structures on the apical surface of ChPE cells. (b) Microvilli length of ChPE cells in *Atp7b*^*-/-*^ mice were significantly reduced compared to the WT mice; n = 3. Brightfield images were analyzed using ImageJ and evaluated using unpaired two tailed t-test. p ≤ 0.05 was considered statistically significant. *** p ≤ 0.001. (c) Immunofluorescence staining for non-muscle myosin Myh10 (green) on ChPE cells from Atp7b -/- and wild type (+/+) mice. Scale bar; 20 μm. (d) Transmission Electron Micrograph (TEM) of ChPl cells showing altered apical microvilli in 4 weeks old *Atp7b*^*-/-*^ mice compared to the wild type mice. Low (6000 X) and high (20000 X) magnification of choroid plexus epithelial cells show elongated healthy apical microvilli (left panel), whereas in *Atp7b*^*-/-*^ the apical microvilli and ciliary structures are regressed and disorganized (marked with arrows) (right panel). (e) Immunofluorescence staining for cilia with Acetylated tubulin (green) and basal body marker Ƴ tubulin (red) on ChPE cells from *Atp7b*^*-/-*^ and wild type (+/+) mice. Scale bar; 20 μm. Cilia structures were marked with an arrow in the magnified images. (f) Immunofluorescence staining for ciliary membrane marker Arl13b (green) and the basal body marker Ƴ tubulin (red) on ChPE cells from *Atp7b-/-* and wild type (+/+) mice. Scale bar; 10 μm. (g) Percentage of ciliated epithelial cells is decreased in choroid plexus of 4 weeks old *Atp7b-/-* mice compared to the wild type. Data represented as Means ± SD. *P < 0.001 (Welch corrected *t*-test). n = 3.

To identify potential reasons for the abnormal ChPl function, we examined ChPl in more detail. First, we noticed changes in *Atp7b*^*-/-*^ ChPl fine morphology in comparison to control mice (**Fig 4A**). The membrane protrusions, microvilli and cilia, are important morphologic features of ChPl as they increase area of transport and facilitate CSF movement; these protrusions were greatly reduced in number and length in *Atp7b*^*-/-*^ ChPl (**Fig 4B**).

In the epithelial cells, maintenance of membrane protrusions depends on myosins, which regulate dynamics of actin bundles [32]. Immunostaining for myosin 10 known to be involved in filopodia formation and cell adhesion [33] revealed significant changes in its intracellular distribution. In control ChPl cells, myosin 10 was uniformly concentrated in the vicinity of plasma membrane, whereas in *Atp7b*^*-/-*^ ChPl the myosin pattern was disorganized with a weaker staining at the apical membrane and a stronger staining towards the basolateral membrane (**Fig 4C**). The transmission electron microscopy (TEM) confirmed disorganization of microvilli in *Atp7b*^*-/-*^ ChPl epithelia (**Fig 4D**). To determine whether Cu misbalance alters abundance of cilia, we used immunostaining for acetylated tubulin (Fig 4E), which revealed fewer cilia in *Atp7b*^*-/-*^ ChPl(**Fig 4E**); this result was confirmed using cilia-specific marker Arl13b (**Figs 4F, 4G** and **S2**)

### Morphological changes in *Atp7b*^*-/-*^ ChPl are associated with significant remodeling of cytoskeleton

**Fig 5:**
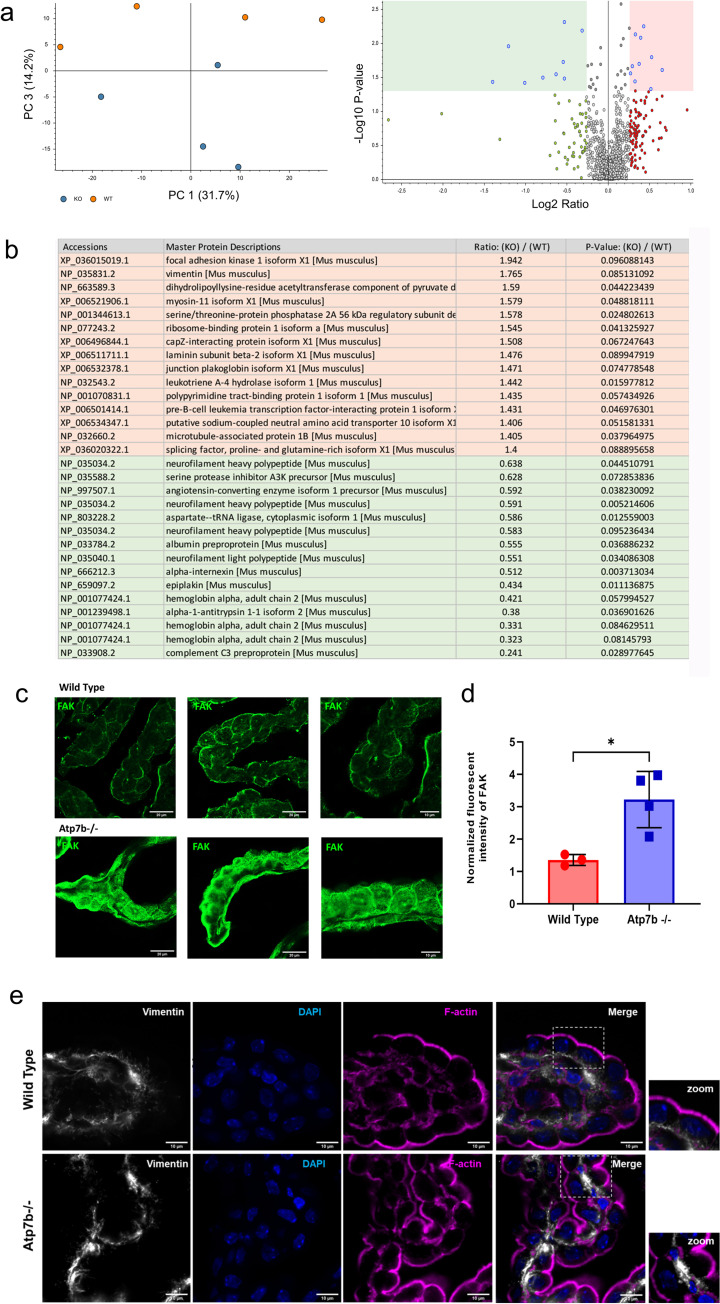
ChPl proteome identifies cytoskeletal changes in Atp7b ^-/-^ mice. **(a)** PCI plot showing clear segregation of ChPl proteome from 4 weeks old Atp7b ^-/-^ and control (n = 4 for each genotype. Volcano plot illustrates the protein distribution. Significantly up- and down-regulated (|fold change| ≥1.3, x-axis; p value≤0.05, y-axis) are highlighted in peach and green, respectively. (b) Table showing significantly upregulated and downregulated proteins in Atp7b ^-/-^ ChPl identified from mass-spectrometry studies. (c) Immunofluorescence staining for focal adhesion kinase (green) on ChPE cells from Atp7b -/- (lower) and wild type (+/+) (upper) mice. Scale bar; 20 μm. (left and middle panel); Scale bar; 10 μm (right panel) (d) Total fluorescence intensity of Focal Adhesion Kinase (FAK) (green) was measured from the confocal images of 4 weeks old wild type and Atp7b-/- ChPl using ImageJ software and normalized to the fluorescent intensity of F-actin (magenta). The data were analysed using two-tailed unpaired t-test with Welch’s correction in Graphpad Prism (9.4.0 software). *P-value; <0.05. (e) Immunofluorescence staining for vimentin (white) and F-actin (magenta) on ChPE cells from Atp7b -/- (lower) and wild type (+/+) (upper) mice. Scale bar; 10 μm.

To better understand the molecular basis of these changes we compared ChPl proteomes in control and *Atp7b*^*-/-*^ mice. For quantitative comparison, identical areas of ChPl were laser-dissected from the 4-weeks-old control and *Atp7b*^*-/-*^ brain sections, and the protein profiles were analyzed by mass-spectrometry following TMT labeling. The PCI plot showed clear separation of control and *Atp7b*^*-/-*^ ChPl proteomes, indicative of reproducible Atp7b-dependent changes (**Fig 5A**). Out of 995 proteins commonly present in all samples, the majority of proteins had similar abundance or showed statistically non-significant changes (**Fig 5B**); 68 proteins showed significant changes (fold change >1.3, p-value <0.1). These latter proteins regulate cell-cell contacts and cell adhesion, mitochondria function, RNA processing, and ion balance (**S1 Table**). The most significantly altered proteins (p-value <0.05)—all—were cytoskeletal proteins and proteins involved in cell-cell contacts. Abundances of focal adhesion kinase, myosin 11, vimentin, junction plakoglobin, tight junction protein 2, plectin, consortin and talin 1 were increased 1.3–2.5-fold (**Fig 5B**). By contrast, actin, hook microtubule tethering protein, epiplakin, a protein that connects intermediate filaments, and myosin 9 were significantly down-regulated along with the heavy and light neurofilament proteins (**Fig 5B and S1 Table**).

To validate findings from mass-spectrometry, we immunostained focal adhesion kinase (FAK) and the type-III intermediate filament, vimentin, both of which were more abundant in *Atp7b*^*-/-*^ proteome. The immunostaining for FAK showed stronger signal in *Atp7b*^*-/-*^ ChPl compared to the age matched controls, confirming proteomics findings (**Figs 5C, 5D and S3**). The pattern vimentin was significantly changed in response to Atp7b inactivation. *Atp7b*^*-/-*^ cells had brightly stained vimentin aggregates in contrast to fine filamentous structures in wild type ChPl (**Fig 5E**). The abundance of vimentin was difficult to compare because of differences in its intracellular distribution. Taken together these results show that the loss of Atp7b is associated with significant remodeling of ChPl cytoskeleton and the protein machinery involved in cell-cell contacts. Changes in cytoskeleton along with downregulation of proteins involved in membrane protein tethering and vesicle trafficking may contribute to inability of Atp7a to traffic to the apical membrane.

### Cu deficit in brain parenchyma does not affect DBH expression

**Fig 6:**
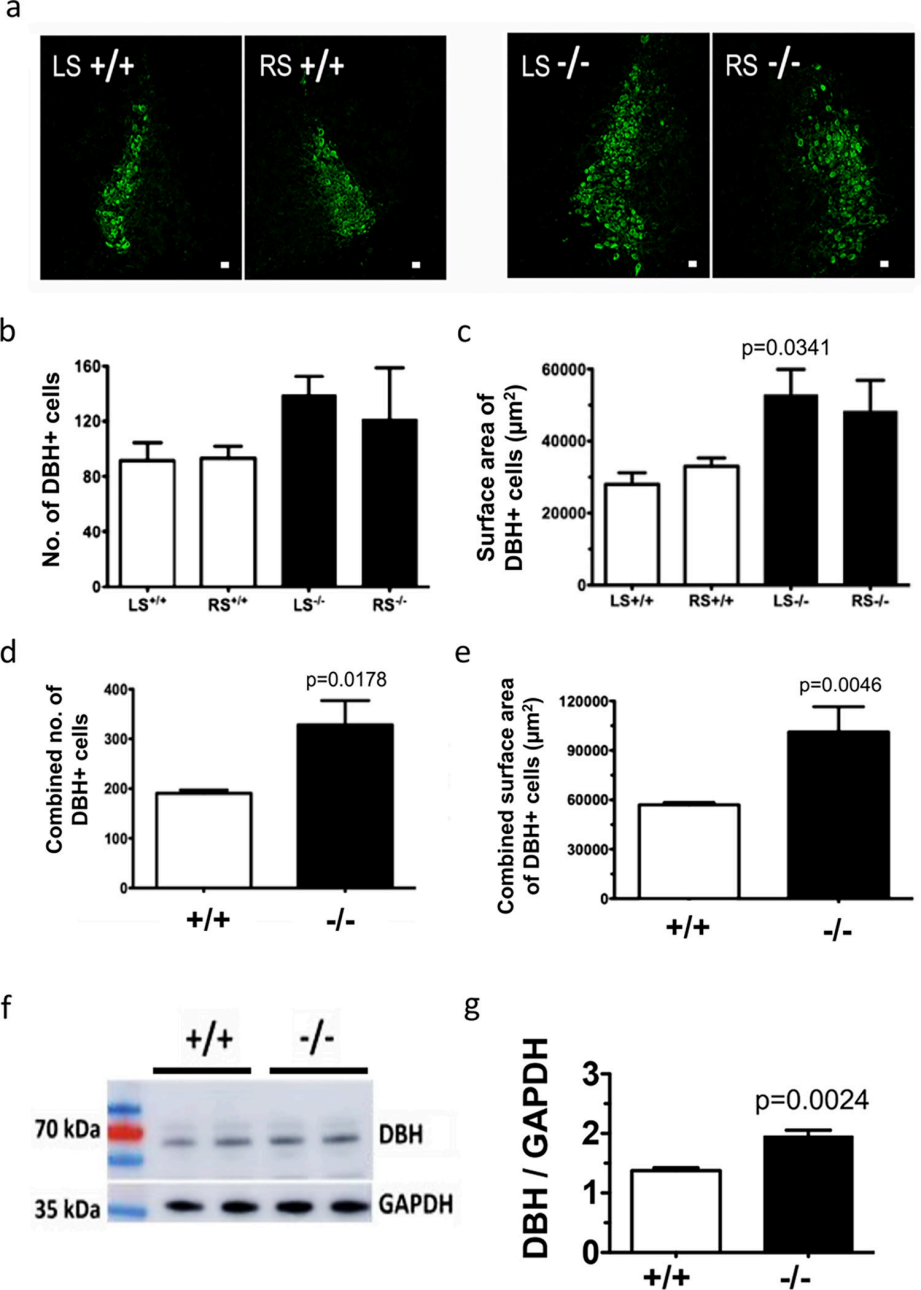
**Inactivation of Atp7b does not decrease DBH expression in mouse brain** (a) Immunofluorescence staining for DBH shows enriched DBH positive cells (DBH+) in the locus coeruleus (LC) region in coronal sections of mice brain tissues. Scale bar, 20 μm. The (b) number and (c) area occupied by DBH+ cells in the left (LS) and right side (RS) of LC were compared between Atp7b-/- and WT (+/+) mice separately. The (d) total number of DBH+ cells and the (e) area occupied by the cells in LC region were compared between Atp7b-/- and WT mice. The data were analyzed using unpaired two tailed t-test. p ≤ 0.05 was considered statistically significant. (f) DBH protein (70 kDa) expressions were compared between *Atp7b*^*-/-*^ and WT (+/+) mice brain using western blot. Expression of housekeeping gene GAPDH was considered as loading control. (g) Densitometric analysis of DBH bands compared to the corresponding GAPDH bands between Atp7b-/- and WT mice. (n = 4) The values represent the mean ± SD in triplicate. p ≤ 0.05 was considered statistically significant. Data analysis was performed using GraphPad Prism 9 software.

To determine whether the ChPl disorganization and a lower Cu content in brain parenchyma have metabolic consequences in the brain, we examined the status of norepinephrine (NE) biosynthesis, which is a Cu-dependent process. The rate-limiting step in this process is the conversion of dopamine (DA) to NE; this step is mediated by the Cu-dependent enzyme dopamine-β-hydroxylase (DBH) [34]. Given that the Cu deficit may cause neuronal death [35], we first examined the number of DBH-positive (noradrenergic) neurons. DBH is highly expressed in locus coeruleus [10], the major site of NE biosynthesis in the brain [36], and DBH immunostaining can be used for identification of LC in tissue sections (**Fig 6A**). The DBH-positive area in *Atp7b*^*-/-*^ sections appeared enlarged (**Fig 6A**). Therefore, to ensure that the equivalent LC regions were compared between the WT and *Atp7b*^*-/-*^ brains, serial sectioning was carried out starting at 300-microns from the complete closure of the fourth ventricle. Each section was collected at 10 microns thickness and every 5^th^ section was stained for DBH. The DBH-positive cells on both sides of the fourth ventricle were quantified (**S4 Fig**). The sections with the maximum number of DBH-positive cells were used for further analyses.

Counting the DBH-positive cells separately for the left or right LC yielded a slightly higher cell number for *Atp7b*^*-/-*^ LC compared to control, although the difference did not reach statistical significance (**Fig 6B**). DBH-positive cells also appeared to be less densely packed in the Atp7b^*-/-*^ samples and occupied larger area (**Fig 6C**). When the numbers of cells in both LC areas were combined, the number of DBH-positive cells as well as the area occupied by these cells were significantly higher in the *Atp7b*^*-/-*^ LC compared to controls (**Fig 6D and 6E**). The DBH signal intensity was similar in *Atp7b*^*-/-*^ and control brain sections. Western blot analysis of the whole brain lysates also found no decrease in DBH protein levels (**Fig 6F**). In fact, when normalized to GAPDH, a small but statistically significantly increase in DBH protein abundance was detected in *Atp7b*^*-/-*^ brains (**Fig 6G**). Thus, neither the number of DBH positive neurons nor DBH expression in the developing brain are negatively affected by Atp7b inactivation and Cu deficit.

### Atp7a, dopamine (DA) and norepinephrine (NE) levels are low in the locus coeruleus of 4 weeks-old *Atp7b*^-/-^ mice

**Fig 7:**
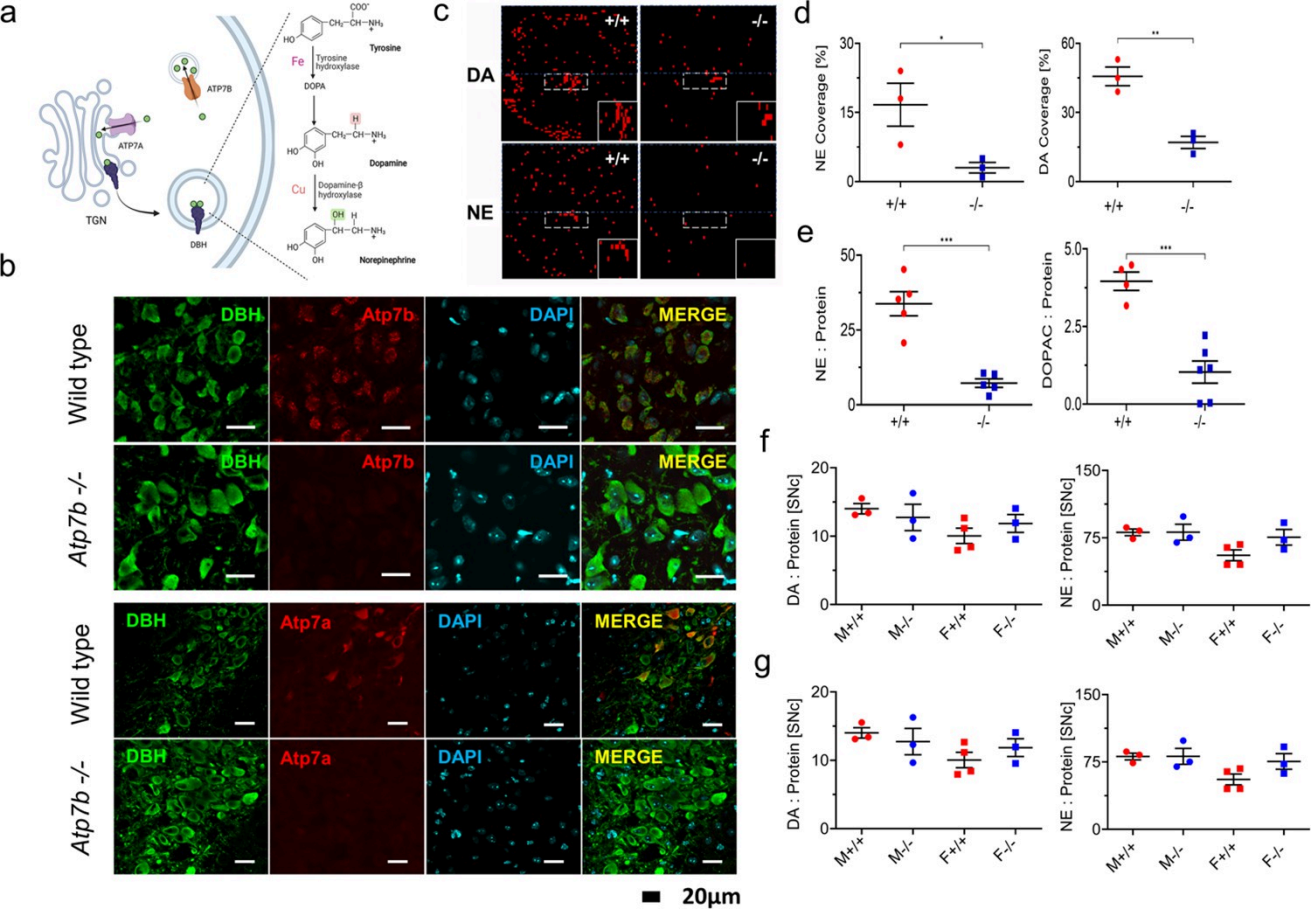
Dopamine (DA) and norepinephrine (NE) are reduced in 4 weeks old Atp7b -/- mice. (a)Schematic diagram showing the role of dopamine β hydroxylase (DBH) in DA to NE conversion. The Cu transporter Atp7a delivers Cu to DBH, while Atp7b buffer cytosolic Cu. (b) Immunofluorescence staining shows Atp7b (red) expression in DBH-positive neurons of control locus coeruleus (LC). No staining for Atp7b is detected in tissues from Atp7b-/- mice. Atp7a (red) is expressed in the LC of control mice and down-regulated in the age-matched Atp7b-/- LC. (c)The DA and NE signals were compared between Atp7b -/- (n = 9) and wild type (+/+) mice (n = 8) in the LCcontaining region. (d) The signal density for NE and DA was determined as a ratio between signal containing pixel to the total area. The data were analyzed using unpaired two tailed t-test. p ≤ 0.05 was considered statistically significant. (e) NE and DA metabolite 3,4-dihydroxyphenylacetic acid (DOPAC) levels in brainstem homogenates were measured using HPLC. p ≤ 0.05 was considered statistically significant. (f) DA and NE levels from substantia nigra (SNc) and (g) striatum (Str) were compared between Atp7b -/- and wild type (+/+) mice. All the values for DA, NE and DOPAC were normalized to the total protein. M, Male; F, Female. Data analysis was performed using GraphPad Prism 9 software.

Cu delivery to DBH is mediated by Atp7a, whereas Atp7b sequesters Cu into vesicles to buffer cytosolic Cu (**Fig 7A**) [18]. Loss of Atp7a function reduces the NE biosynthesis and disrupts LC function [37]. Immunostaining of control tissue sections showed that both Atp7a and Atp7b were expressed in the LC neurons at 4 weeks after birth **(Fig 7B)**, although at a lower level and less uniformly than in the adult brain (**S2 Fig**). Atp7a abundance was significantly reduced in 4 weeks old *Atp7b*^*-/-*^ LC compared to control LC (**Fig 7B**). Thus, the major route of Cu delivery to DBH–via Atp7a protein–was compromised in *Atp7b*^*-/-*^ mice, in addition to overall low Cu levels in brain parenchyma. These results suggested that the NE production was impaired in *Atp7b*^*-/-*^ LC. To test this hypothesis, DA and NE were derivatized in control and *Atp7b*^*-/-*^ brain tissue sections and imaged using matrix assisted laser desorption/ionization mass spectrometry (MALDI-MSI); the identities of derivatized DA and NE in sections were confirmed by collision-induced dissociation (**S4 Fig**)

MALDI-MS imaging of DA and NE revealed significant differences in catecholamine levels in *Atp7b*^*-/-*^ brains compared to control (**Fig 7C**). Signal density, determined as a ratio of signal-containing pixels to the total area, was significantly lower in *Atp7b*^*-/-*^ sections–for both DA and NE. Quantitation of the DA and NE signals per area more specifically within the LC-containing region (**Fig 7C**; dotted white box) found a 2.5-fold decrease of the DA signal and an 80% decrease of the NE signal in *Atp7b*^*-/-*^ mice compared to control mice (**Fig 7D**). To verify these changes in catecholamine levels, especially the decrease in NE, the brainstem region (the part of the brain where LC is located) was dissected, and catecholamines levels were measured by HPLC_ECD (**Fig 7E**). The levels of NE were lower in *Atp7b*^*-/-*^ samples when compared to controls in agreement with the imaging data. Dopamine metabolite, DOPAC, was also lower reflecting the diminished DA levels in *Atp7b*^*-/-*^ samples (**Fig 7E**)

In contrast to a brainstem, no statistically significant difference in the DA and NE levels was found in the substantia nigra region (**Fig 7F**). The DA turnover ratio was slightly elevated, but this difference was not statistically significant (**S5A–S5C Fig**). In striatum, the mean levels of DA were lower in the ko males compared to controls, whereas changes in NE levels were not statistically significant (**Fig 7G**). Sex-specific differences were observed in control animals: control males had highest DA levels in both substantia nigra and striatum (**Fig 7G**). The DA and NE levels were both lower in the substantia nigra of control females compared to males. In contrast, no sex-dependent differences in either DA or NE was seen in *Atp7b*^*-/-*^ substantia nigra (SNc) (**Fig 7F**). Taken together, these data illustrate that catecholamine biosynthesis is altered in *Atp7b*^*-/-*^ brain at 4 weeks, especially in the LC/brainstem region.

### Inactivation of Atp7b causes specific changes in brain lipidome

**Fig 8:**
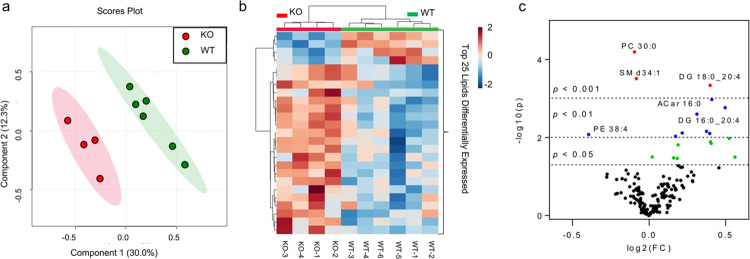
**Changes in Atp7b-/- brain lipid profile at 4 weeks after birth compared to control** (a) Partial Least Squares–Discriminate Analysis (PLS-DA) plot comparing WT (green) and KO (red) in positive ion mode LC-MS/MS; R2 = 0.99, Q2 = 0.48. Each point represents a data set from an individual animal. The 95% confidence intervals are indicated by elliptical shaded areas surrounding each group. Data were sum normalized, log transformed, and mean centered. (b) Heatmap displaying the top 25 differential abundance features based on t-test, Euclidean distancing, and ward clustering in positive ion mode LC-MS/MS. (c) Volcano plot highlighting features that had a p < 0.05 (green), p < 0.01(blue), and p < 0.001 (red) when comparing WT to KO. Selected lipids are indicated. The x-axis is log2 (FC) (FC = fold change) and the y-axis is–log10(p) (p = p-value based on t-test). Acar = acylcarnitine, DG = diacylglycerol, PC = glycerophosphocholine, and SM = sphingomyelin. Plots in A-C generated using Metaboanalyst; n = 6 for WT, n = 4 for KO.

Given changes in ChPl morphology in *Atp7b*^*-/-*^, which were indicative of decreased epithelial surface, we tested whether other metabolic processes that do not directly depend on Cu (such as lipid metabolism) were affected in *Atp7b*^*-/-*^ brain. Lipids were extracted from the whole brain and analyzed by liquid chromatography coupled to high resolution mass spectrometry (HRMS). Partial Least Squares–Discriminate analysis (PLS-DA) of the datasets revealed close clustering of samples within the genotypes (i.e., control and *Atp7b*^*-/-*^) and significant difference between the genotypes, indicative of changes in lipidome in response to Atp7b inactivation (**Fig 8A**). The heat map of top 25 most significantly different lipids shows inversely correlated lipid profiles in control and *Atp7b*^*-/-*^ mice (**Fig 8B**), which suggested potential precursor-product relationship in lipid synthesis. To test this hypothesis, we identified the most significantly changed lipids using volcano plot (**Fig 8C**). Phosphatidylethanolamine (PE) levels were significantly reduced in the *Atp7b*^*-/-*^ brain when compared to the age-matched control mice, whereas levels of diacylglycerol DG 18:0_20:4, a precursor of PE, were elevated (**Fig 8C**). Ethanolamine phosphotransferase 1 (EPT1) is the Golgi-localized enzyme that has substrate specificity for DG 18:0_20:4, converting it into PE [38]. Accumulation of the substrate and reduction of the product suggested that the EPT1 activity was decreased in the developing *Atp7b*^*-/-*^ brain.

In summary, during postnatal brain development inactivation of Atp7b causes significant changes both at the blood-CSF barrier and in brain parenchyma, including region-specific alterations in catecholamine levels, dysregulation of lipid metabolism, and remodeling of cytoskeleton in ChPl. These changes occur long before Cu accumulation and reflect important role of Atp7b in the intra- and inter-cellular Cu distribution during postnatal brain development.

## Discussion

Choroid plexus plays a central role in the maintenance of CSF ion balance and the delivery of micronutrients to brain parenchyma. Here, we demonstrate the importance of Atp7b-dependent Cu homeostasis for ChPl morphology and function. The multifaceted impact of Atp7b inactivation on ChPl and brain parenchyma during brain development offers new insight into Wilson disease (WD) pathogenesis. In WD, Cu accumulates most highly in the liver, where it causes alterations of mitochondria function, dysregulation of nuclear receptor signaling, inflammatory response, and other pathologies [5]. Cu accumulation in the brain occurs later, when the liver capacity to accumulate Cu is saturated [22,24]. It is thought that metabolic changes in the brain are triggered by excess Cu released from a necrotic liver later in WD progression [5]. Here, we show that, in *Atp7b*^*-/-*^ mice, brain metabolism is dysregulated as early as at 4 weeks after birth and these changes are caused by transient Cu deficiency, which is only later replaced by Cu accumulation. Transient Cu deficit in brain parenchyma is associated with upregulation of Atp7a in ChPl and down-regulation of Ctr1 at the apical membrane of ChPl epithelial cells. These changes may represent a protective response aimed to increase/retain brain Cu content. Significantly, the dysregulated metabolic pathways involve those directly requiring Cu (NE biosynthesis) as well as those indirectly affected by tissue Cu content (lipid metabolism). It is tempting to speculate that metabolic changes or environmental stresses that impact these important pathways may accelerate development of neuropathology in WD. Excessive Cu chelation could be one of such stressors.

Given that Atp7a is primarily responsible for Cu entry into the brain and the expression of Atp7a is not decreased in *Atp7b*^*-/-*^ ChPl, the observed Cu deficit in brain parenchyma may seem surprising. The origin of this Cu deficiency could be two-fold. Although the exchangeable Cu is not decreased in the serum of young *Atp7b*^*-/-*^ mice [39] and organs other than brain accumulate Cu (Fig 1), we cannot exclude that in young mice less Cu enters ChPl at the basolateral side. In humans, Ctr1 malfunction causes marked Cu deficit in the brain, and it has been proposed that Ctr1 transfers Cu into ChPl through the basolateral membrane [40]. Our data neither support nor disprove this model. In 4 weeks-old mice, the Ctr1 signal at the basolateral aspect of epithelial cells is more consistent with the staining of stroma rather than plasma membrane, but further studies are needed to firmly determine whether Ctr1 is targeted and functions at the basolateral membrane. Similarly, the resolution and sensitivity of our LA-ICP-MS measurements are insufficient to accurately determine the Cu content in ChPl and further high-resolution studies are needed. Indeed, at 20 weeks, when *Atp7b*^*-/-*^ liver takes less Cu [41,42], more Cu is available for the brain and Cu begins to accumulate in the *Atp7b*^*-/-*^ brain tissue.

Another factor that may limit Cu entry into the brain is significant reorganization of cytoskeletal elements, which may impact protein trafficking to the plasma membrane. Related phenomenon was observed in the intestine, where downregulation of Atp7b causes decrease in the total intestinal Cu content, abnormal protein trafficking and disrupted iron efflux [43]. In both intestine and ChPl, the expression of Atp7a is not compromised, instead the pathways for protein transport and delivery to plasma membrane are dysregulated. In intestine, the intracellular Cu misbalance alters trafficking of ApoB and chylomicrons assembly [43]; in ChPl, there is significant remodeling of cytoskeleton, which impacts the number and length of cilia and may influence the ability of Atp7a to reach the plasma membrane, although this remains to be formally tested. It is interesting that Atp7b inactivation in mice affects the abundance of neurofilaments, because recent clinical studies found serum neurofilament light chain as a biomarker of neurologic WD [44]. Further studies are needed to better understand the relationship between cytoskeletal changes in ChPl, axonal health and serum neurofilaments in human WD and in WD models.

Another fascinating question is whether changes of cytoskeleton or other Cu-dependent processes are responsible for reduced number of cilia. Peptidyl-alpha-monooxygenase (PAM) is an important Cu-dependent enzyme, which is required for the normal assembly of motile and primary cilia [45]. PAM is expressed in ChPl [46] and its reduced expression was shown to be associated with reduced density of motile cilia [45]. Changes in cytoskeleton may also alters release of PAM and neuropeptide-containing exosomes [47] further altering signaling events.

Changes in catecholamine balance were reported in WD patients and animal models of WD [48–50]. These studies showed significant variability of specific changes in the DA and NE levels depending on the age and brain region [48]. Our results agree with these observations. As in rats, we see decrease in NE prior to Cu accumulation. Atp7b inactivation causes reduction of DA and NE levels in the LC region of the brainstem, whereas in other regions of the brain NE levels were not significantly affected and the DA levels were less significantly changed. Low abundance of Atp7a in the LC (where the bodies of noradrenergic neurons are located) may explain stronger negative effect on the NE content compared to other regions. Whether Atp7a redistributes from the cell bodies to neuronal projections to maintain DBH activity and NE synthesis locally remains to be tested. Some decrease in Fe may contribute to DA deficit, as the conversion of tyrosine to DOPA (precursor of dopamine) is mediated by Fe-dependent tyrosine hydroxylase, however later in the disease Fe levels are normalized (at least in the brain) and other mechanisms effecting dopaminergic neurons must be at play.

Lipid metabolism is dysregulated in response to either Cu overload [51–54] or Cu deficiency as observed in Menkes disease [55]. Our data illustrate that Atp7b activity is required for formation of phosphatidylethanolamine in the developing mice brain. Low levels of phosphatidylethanolamine are indicative of low methylation of PC to form PE. Future studies are needed to determine whether inhibition of diacylglyceride-to-phosphatidylethanolamine conversion could be caused by changes in availability of methyl donors as was reported for the liver in another model of WD (tx mice [56,57]).

In conclusion, our results illustrate a previously unappreciated role of Atp7b during brain maturation and indicate that WD pathogenesis is more complex than tissue response to Cu overload. In mice, functional maturation of the brain is completed within 35–50 days after birth, with the peak of myelination and specialization of the prefrontal cortex neural networks occurring at 3–4 weeks after birth [58]. In humans, similar brain maturity is achieved at 12–18 years after birth. The lack of obvious neurologic symptoms in mice and most pediatric WD patients led to the assumption that ATP7B inactivation does not affect brain metabolism prior to Cu overload.

Our findings illustrate that the significant metabolic changes may occur when the total Cu in the brain is transiently low or not yet unchanged. Our data also suggest that the side-effects of Cu chelation (such as neurologic worsening and GI discomfort) may be caused by tissue-specific worsening of local/transient Cu deficits during treatment.

## Materials and methods

### Ethics statement

All animal experiments were performed in accordance with the protocol (M017M385) approved by the Johns Hopkins University Institutional Animal Care and Use Committee.

### Mice husbandry and tissue collection

The C57BL/6J mice and the B6;129S1-*Atp7b*^tm1Tcg^/LtsnkJ (*Atp7b*^*-/-*^) mice on a C57BL/6J background [59] were used in this study. These stains will be referred to as control and *Atp7b*^*-/-*^ mice (knockout), respectively, throughout the manuscript. Mice were housed at Johns Hopkins School of Medicine Animal facility; the experiments were performed in accordance with the protocol (M017M385) approved by the Johns Hopkins University Institutional Animal Care and Use Committee. At 4 weeks or 20 weeks after birth, mice were euthanized, perfused with the phosphate buffered saline (PBS) and then for 2 min with 4% paraformaldehyde (PFA) solution. Whole brain was extracted and stored in a 4% PFA solution overnight at 4°C. Then, brains were transferred to a 30% sucrose solution overnight at 4°C, embedded in Tissue-Tek OCT freezing medium, and stored at– 80°C for cryo-sectioning. Alternatively, after PBS perfusion, the whole brain was frozen using dry ice.

For metal analysis, brain was perfused with phosphate buffered saline (PBS) and then with 4% paraformaldehyde (PFA) solution as above. Metal levels were measured using whole brain (**Fig 1A** top panels) as well as liver, kidney, spleen, and heart (**Fig 1B**). Weighed samples were digested in nitric acid and analyzed by atomic absorption spectrometry (Perkin Elmer Pinnacle 900T). Metal content was quantified and divided by tissue weight.

### Cryo-sectioning and sections selection

Brains frozen in OCT were removed from– 80° C, equilibrated to– 20° C and sectioned from the brainstem towards the prefrontal cortex using Leica Cryostat CM 3050S. Ten-micron coronal and sagittal sections were placed onto Superfrost Plus microscope slides (Thermo: Cat. No. 12-550-15), two sections per slide. Every fifth slide was stained using anti-rabbit dopamine-β-hydroxylase (DBH) primary antibody (kindly provided by Dr. Betty Eipper) or anti-rabbit tyrosine hydroxylase (Millipore; Cat. No. AB152) as described below, and immunofluorescence was imaged using Zeiss LSM800 confocal microscope. Images were imported into ImageJ, and DBH-positive cells and the area occupied by these cells were quantified. The values were plotted using GraphPad Prism version 5 software. Sections with the highest number of DBH-positive cells were used for further analysis.

### Immunohistochemistry of tissue sections

Brain sections were removed from– 80° C and washed with 1X PBS to remove residual OCT medium. The primary antibodies were diluted in 1X PBS containing 0.5% BSA (Bovine Albumin Serum, Millipore; CAS. No. 9048-46-8), 0.1% Tween-20 (Sigma Aldrich; CAS. No. 9005-64-5), and 0.5% Triton X-100 (Sigma Aldrich; CAS. No. 9002-93-1). DBH primary antibody at 1:1000 dilution was added and incubated for 1 hour at room temp or 4° C overnight. Brain sections were then washed three times in 1X PBS for 5 minutes. Goat anti-rabbit Alexa Fluor 488 (Invitrogen; Cat. No. A-11008) or Alexa Fluor 555 (Invitrogen; Cat. No. A-21434) were diluted into 1X PBS containing 0.5% BSA and 0.1% Tween-20. The secondary antibody was added at 1:1000 for 3 hours at room temperature. Brain tissue sections were then washed three times in 1X PBS for 5 minutes. Fluoromount-G with DAPI (Electron Microscopy Sciences 17984–24) was used to mount a 24 x 50 mm coverslip onto the microscope slide. Slides were kept in the dark for one hour and then imaged using either the Zeiss LSM800 or Olympus FV3000RS microscopes. Anti-ATP7A and anti-ATP7B primary antibodies were purchased from Santa Cruz (SC-376467 and SC-373964, respectively). Anti-NKCC1 antibody was purchased from Cell Signalling (D208R XP; 85403). Anti Arl13b, Anti gamma tubulin and Anti acetylated tubulin antibodies were purchased from Proteintech (17711-1-AP), Milllipore-Sigma (T6557) and Cell Signalling (D20G3 XP; 5335) respectively from Anti-Focal Adhesion Kinase (FAK) and Anti-Vimentin antibody were purchased from Invitrogen (MA5-15588) and Abcam (ab92547) respectively. Myosin heavy chain 10 (MYH10) primary antibodies were purchased from Invitrogen (Cat no. MA5-27767). F-actin was stained with Phalloidin AlexaFluor 647 (Invitrogen, A22287). All antibodies were used in accordance with the procedures described above.

### Transmission Electron Microscopy

ChPl tissue was freshly isolated from the brain ventricles of 4 weeks old *Atp7b*^*-/-*^ and wild type mice and fixed by immersion with EM grade 2% paraformaldehyde 1% glutaraldehyde 100 mM phosphate buffer (Sorenson’s) and 3 mM magnesium chloride pH 7.2 at 4°C. all subsequent steps were done at 4°C until 70% ethanol dehydration. Tissues were dissected carefully in fixative measuring no more than 2 mm^3^. Samples were rinsed in buffer containing 3% sucrose (3 X 15 min). Osmication was performed in 1.5% potassium ferrocyanide reduced 1% osmium tetroxide in 100 mM phosphate buffer, containing 3 mM magnesium chloride for 2 hrs at 4°C. Tissue was rinsed in 100 mM Maleate buffer (3 X 5 min) containing 3% sucrose, then en-bloc stained with 2% filtered uranyl acetate in the same buffer for 1 hr. Samples were dehydrated to 100% ethanol.

Propylene oxide (2 X 5 min) was used as a transition solvent, and the tissue was embedded with eponate 12 (Ted Pella, Redding CA) and finally cured in 60°C oven for 2 days, then sectioned to 60 nm using diamond microtome. EM images were collected using a Hitachi 7600 transmission electron microscope operated at 80.0 kV. Images were acquired at 6,000 to 20,000 × direct magnification, corresponding to 0.010744 μm/pixel to 0.003223 μm/pixel, respectively.

### Derivatization, matrix application, and MALDI mass spectrometry imaging (MSI)

MALDI matrix, α-cyano-4-hydroxycinnamic acid (CHCA) was obtained from Millipore Sigma (USA). All solvents were purchased from Fisher Scientific (USA), unless otherwise specified and were either reagent or high-performance liquid chromatography (HPLC) grade. TMP (2,4,6-trimethyl pyrylium) solutions were prepared as previously described [60]. TMP stock solution (7 mg in 3 mL methanol) was diluted in 4 mL of 70% methanol containing 3.5 μL triethylamine. The diluted TMP was sprayed over the tissue sections using a TM-Sprayer (HTX Technologies, LLC, Chapel Hill, NC) as described previously [61]. After 15 min incubation in a chamber saturated with vapor from 50% methanol, a 5 mg/mL CHCA (Sigma-Aldrich, USA) in 50% acetonitrile containing 0.2% TFA was applied to the tissue sections using a TM-Sprayer (HTX Technologies, LLC, Chapel Hill, NC) as described previously [62]. All MALDI MSI experiments were performed using an LTQ Orbitrap XL (Thermo Fisher Scientific, Bremen, Germany) mass spectrometer in positive ion mode with a direct beam N_2_ laser (λ = 337.7 nm). A mass range of 100 to 1000 Dalton was used for data acquisition to cover the *m/z* values of DA and NE. The mass spectrometry data processing and analysis were carried out using Xcalibur 3.0 software (Thermo Fisher Scientific). For tissue imaging experiments, the MALDI camera was employed to define regions of interest by scanning the slides containing the tissue sections. Distribution profiles of DA and NE were generated at a spatial resolution of 100 μm using Image Quest 1.1.0 software (Thermo Fisher Scientific). For calculation of relative DA and NE levels (based on the coverage); the total number of signal pixels was divided per total area pixels”. The percentage of coverage was then compared.

### HPLC quantification of norepinephrine in the brainstem containing LC region

Neurotransmitter concentrations were measured by HPLC-ECD, as previously described [63]. Briefly, mice were euthanized by decapitation and the brain was frozen at -80C until brainstem microdissection. Tissue was weighed and sonicated in 0.2 μl of ice-cold 0.01 μM perchloric acid containing 0.01% EDTA and 60 nM 3,4-dihydroxybenzylamine as an internal standard. After centrifugation at 15,000 g, 4°C for 30 min, the supernatant was passed through a 0.2-μm tube filter. Twenty microliters of the supernatant were analyzed in an HPLC column (Atlantis T3, 3μM × 150 μM C-18 reverse-phase column; Waters, Milford, MA, USA) with detection by a dual-channel Coulchem III electrochemical detector (Model 5300; Thermo Fisher Scientific). Protein concentrations in the tissue homogenates were measured using the BCA Protein Assay Kit (Thermo Fisher Scientific). Data were normalized to protein concentrations (ng neurotransmitters/mg protein).

### Copper imaging using laser ablation inductively coupled plasma mass spectrometry

PBS perfused mouse brains were dissected and immediately embedded in Optimal Cutting Temperature (OCT) mounting media (Tissue Tek) in cryomolds (Tissue Tek), then flash-frozen in a liquid nitrogen/isopentane bath and stored at -80° C until sectioning. The embedded brains were sectioned into 20 μm slices using a (Leica Cryostat CM 3050S). The slices were air-dried and stored at room temperature until analysis. Laser ablation was performed on an NWR213 laser with a TV2 sample chamber (ESI, Bozeman, MT) using the following parameters: Spot size: 6 μm; Fluence: 3.1 J cm-2; Stage speed: 15 μm s-1; Firing rate: 20 Hz; He flow: 800 mL min-1; Pattern spacing: 6 μm. The ablated material was introduced by Helium flow into an iCAP-Qc ICP-MS (Thermo Fisher) and analyzed for 63Cu using a 0.4 sec dwell time in standard acquisition mode. The resulting mass spectrometry traces and laser log files were processed in Igor Pro using the Iolite application with a custom matrix.

### Laser-mediated dissection of ChPl and proteome analysis

Mouse brains were sectioned at a thickness of 10 μm on a cryostat (Leica CM3050 S) set to a– 20°C and collected on super plus frosted slides (Fisher Scientific, USA). Sections were then stored at– 80°C. For laser capture, the paired control and KO tissues sections were removed from the– 80°C freezer, warmed to room temperature for approximately 30 minutes. Slides were briefly fixed in ice cold ethanol in two steps; first 100% ethanol (2 dips, 3 seconds each), followed by two dips with 70% ethanol. The ethanol was allowed evaporate before two washes in PBS for 2 minutes each. Slides were then washed twice in deionized H_2_O for 30 seconds. Excess water was wiped off the slide’s edges with a Kimwipe and allowed to air dry for about 5–10 minutes. Air dried slides were loaded onto the stage of the Axio Observer A1 portion of the Palm MicroBeam from Zeiss. Using the Palm Robo software (Zeiss), the sections were screened using the transmission mode, a magnification of 5x, 272 millisecond exposure, and a numerical aperture. of 0.17 to locate 4^th^ ventricle. The free hand tool was used to outline the choroid plexus. The LCM was then switched to 20x magnification, and the numerical aperture was increased to 0.22. The sections were then laser-cut and collected on adhesive cap tubes (Zeiss). To collect the same total area of 3.05 mm^2^ 6–16 sections per animal were used. Collected material was incubated for 1 hour in a buffer containing 8M Urea, 50 mM TEAB, 0.444 M beta-mercaptoethanol, and 0.74% (M/V) SDS. After incubation, the samples are centrifuged at 10,000g, and transferred to a fresh Eppendorf tube.

TMT labeling and analysis of the protein extracts were performed by the Johns Hopkins Mass-spectrometry and Proteomics facility (see S1 Text). The identified proteins were sorted based on fold-change and p-values and those with *p*-value less than 0.05 and fold-change more 1.5 were submitted to Ingenuity Pathway Analysis (Ingenuity, Thermo Fisher Scientific, Halethorpe, MD) for functional classification. Additional details can be found in S1 Text.

### Lipid extraction and analysis

Total lipid extracts from 10 mg of homogenized brain tissue were prepared using a modified MTBE lipid extraction protocol [64]; for details see S1 Text. Total lipid extracts were analyzed by liquid chromatography coupled to high resolution mass spectrometry (HRMS). For HPLC analysis see S1 Text. Mass spectrometry analysis was separated into two workflows: 1.) lipid identification of a pooled sample using an iterative MS/MS acquisition and 2.) lipid semi-quantitation of all samples using high-resolution, accurate mass MS1 acquisition. The MS parameters for the iterative workflow were as follows: extended dynamic range, 2 GHz; gas temperature, 200°C; gas flow, 10 L/min; nebulizer, 50 psi; sheath gas temperature, 300°C; sheath gas flow, 12 L/min; VCap, 3.5 kV (+), 3.0 kV (–); nozzle voltage, 250V; reference mass m/z 121.0509, m/z 1221.9906 (+), m/z 119.0363, m/z 980.0164 (–); MS and MS/MS Range m/z 100–1700; acquisition rate, 3 spectra/s; isolation, narrow (~ 1.3 m/z); collision energy 20 eV (+), 25 eV (–); max precursors/cycle, 3; precursor abundance-based scan speed, 25,000 counts/spectrum; ms/ms threshold, 5,000 counts and 0.001%; active exclusion enabled yes; purity, stringency 70%, cut off 0%; isotope model, common organic molecules; static exclusion ranges, m/z 40 to 151 (+,–). The MS parameters for the MS1 workflow were the same for source and reference mass parameters and differed only for acquisition (selection of MS (same parameters) not Auto MS/MS).

LC-MS data from the iterative MS/MS workflow was analyzed for lipid identification via Agilent’s Lipid Annotator (v 1.0). The default settings for feature finding and identification parameters were used. Positive and negative ion mode adducts included [M+H]+, [M+Na]+, [M+NH_4_]+, [M-H]-, and [M+CH_3_CO_2_]-, respectively. The results of the Lipid Annotator were saved as a PCDL file. The LC-MS data from the MS1 workflow were processed using Agilent’s MassHunter Profinder (v 10.0). Batch targeted feature extraction using default parameters and the PCDL file created from Lipid Annotator were used for feature extraction. The processed data generated from Profinder which included peak area and lipid identification was exported into MetaboAnalyst 4.0 [65,66] for multivariate analysis. Univariate analysis was done using Prism 6 (GraphPad, La Jolla, CA). Additional details can be found in in S1 Text)

## Supporting information

S1 Fig **Localization of Ctr1 at the apical membrane and the basolateral (stromal) aspect of ChPl** (a) Immunofluorescent staining for Ctr1 (Red) and apical membrane marker NKCC1 (Green) on ChPl from 4 weeks Atp7b-/- and Wild type. ChPl was co-stained with F-actin (Phalloidin_Alex647; magenta). The Ctr1 shows colocalization with NKCC1 in wild type ChPl on the apical membrane which was completely absent in Atp7b-/-. Ctr1 staining was also observed on the basolateral membrane of ChPl. The magnified area was marked with a white dotted box. The apical membrane has been marked with white arrows. (b) The colocalization of Ctr1 with NKCC1 and F actin was shown using RGB profile plot. The area for which the RGB profile plot was generated has been shown with a white line. Scale bar 20 μm.
(PDF)

S2 Fig **Cilia density is decreased in the 4 week-old Atp7b-/- ChPl compared to controls** (a) Immunofluorescent staining for ciliary membrane marker Arl13b (green), gamma tubulin (red) on ChPl from 4 weeks Atp7b-/- and Wild type controls. The data here for Arl13b/γ-tub/DAPI merged is the same as in Fig 3G. Scale Bar 20 μm (b) Merged Z stack images for Arl13b (white) and gamma tubulin (red) from Wild type and Atp7b-/- ChPl were used to count the number of ciliated cells. The ciliated structures were marked using a white box. Scale Bar 20 μm (c) Percentage of ciliated epithelial cells between Atp7b-/- and wild type ChPl were calculated from these images. Data represented as Means ± SD. *P < 0.001 (Welch corrected t-test). n = 3–4
(PDF)

S3 Fig (extended Fig 4C). **Immunofluorescent staining for focal adhesion kinase (FAK) on ChPl from 4 weeks Atp7b-/- and Wild type controls.** The ChPl was stained for FAK (green), Transthyretin (TTR) (Red), and F-actin (Magenta). The data here for FAK is the same as in Fig 4C. The quantitation for FAK shown in Fig 4D was performed by normalizing the fluorescent intensity of FAK to F-actin. Scale bar 20 μm. S4 Fig. Quantification of DBH-positive cell located at both sides of the fourth ventricle in 4-weeks-old control C57Bl/6 mice. (A) Representative cross sections and DBH staining at various distances from a complete closing of the fourth ventricle. (B) DBH-positive cells on both sides of the fourth ventricle were quantified and plotted using individuate sample replicates. (C) Average of DBH-positive cells at various distances from the complete closing of the fourth ventricle.
(PDF)

S4 Fig Quantification of DBH-positive cell located at both sides of the fourth ventricle in 4-weeks-old control C57Bl/6 mice. (A) Representative cross sections and DBH staining at various distances from a complete closing of the fourth ventricle. (B) DBH-positive cells on both sides of the fourth ventricle were quantified and plotted using individuate sample replicates. (C) Average of DBH-positive cells at various distances from the complete closing of the fourth ventricle.
(PDF)

S5 Fig Expression of Atp7a and Atp7b (red) in DBH positive cells (green) of locus coeruleus at 4 weeks and 20 weeks after birth. The data for Atp7a at 4 weeks here is the same as in the Fig 5B. Scale bar = 20μm.
(PDF)

S6 Fig (top two panels) Representative average full scan mass spectra of derivatized DA and NE in brain tissue exhibiting the detection of their ions at m/z 258.1485 and 274.1435, respectively. CHCA was used as the matrix. (bottom panels) In order to confirm the identities of derivatized DA and NE, collision-induced dissociation was carried out. From these experiments, derivatized DA exhibited fragment ions at m/z 122.0958 and 137.0591 corresponding to C8H12N+ and C8H8O2+, respectively. Similarly, derivatized NE yielded its fragment ions at m/z 122.0959 and 153.0540 corresponding to C8H12N+ and C8H8O3+, respectively.
(PDF)

S7 Fig Analysis of dopamine degradation products in striatum (Str) and substantia nigra (SNc) in males and females. 5-HIAA is 5-hydroxyindoleacetic acid, DOPAC—3,4-dihydroxyphenylacetic acid, 5HT—5-hydroxytryptamine, 3MT—3-homovanillic acid (n = 3–4)
(PDF)

S1 Text Supplemental methods and reference. (PDF)

S1 Table Significant changes in protein abundances in Atp7b-/- ChPl compared to control (p-value<0.1). Peach color indicate proteins upregulated more than 1.25 fold; green color–proteins downregulated more than 1.25 fold.
(PDF)

## References

[1] Chang CJ. Searching for harmony in transition-metal signaling. Nat Chem Biol. 2015;11(10):744–7. doi: 10.1038/nchembio.1913 26379012

[2] Monnot AD, Zheng G, Zheng W. Mechanism of copper transport at the blood-cerebrospinal fluid barrier: influence of iron deficiency in an in vitro model. Exp Biol Med (Maywood). 2012;237(3):327–33. doi: 10.1258/ebm.2011.011170 22442359 PMC3982225

[3] Kaler SG, Holmes CS, Goldstein DS, Tang J, Godwin SC, Donsante A, et al. Neonatal diagnosis and treatment of Menkes disease. N Engl J Med. 2008;358(6):605–14. doi: 10.1056/NEJMoa070613 18256395 PMC3477514

[4] Dev S, Kruse RL, Hamilton JP, Lutsenko S. Wilson Disease: Update on Pathophysiology and Treatment. Front Cell Dev Biol. 2022;10:871877. doi: 10.3389/fcell.2022.871877 35586338 PMC9108485

[5] Czlonkowska A, Litwin T, Dusek P, Ferenci P, Lutsenko S, Medici V, et al. Wilson disease. Nat Rev Dis Primers. 2018;4(1):21. doi: 10.1038/s41572-018-0018-3 30190489 PMC6416051

[6] Machado A, Chien HF, Deguti MM, Cancado E, Azevedo RS, Scaff M, et al. Neurological manifestations in Wilson’s disease: Report of 119 cases. Mov Disord. 2006;21(12):2192–6. doi: 10.1002/mds.21170 17078070

[7] Oder W, Grimm G, Kollegger H, Ferenci P, Schneider B, Deecke L. Neurological and neuropsychiatric spectrum of Wilson’s disease: a prospective study of 45 cases. J Neurol. 1991;238(5):281–7. doi: 10.1007/BF00319740 1919612

[8] Starosta-Rubinstein S, Young AB, Kluin K, Hill G, Aisen AM, Gabrielsen T, et al. Clinical assessment of 31 patients with Wilson’s disease. Correlations with structural changes on magnetic resonance imaging. Arch Neurol. 1987;44(4):365–70. doi: 10.1001/archneur.1987.00520160007005 3827691

[9] Lang C, Muller D, Claus D, Druschky KF. Neuropsychological findings in treated Wilson’s disease. Acta Neurol Scand. 1990;81(1):75–81. doi: 10.1111/j.1600-0404.1990.tb00934.x 2330819

[10] Uerlings R, Moreno D, Murillo O, Gazquez C, Hernandez-Alcoceba R, Gonzalez-Aseguinolaza G, et al. Brain copper storage after genetic long-term correction in a mouse model of Wilson disease. Neurol Genet. 2018;4(3):e243. doi: 10.1212/NXG.0000000000000243 29845115 PMC5961192

[11] Dusek P, Litwin T, Czlonkowska A. Neurologic impairment in Wilson disease. Ann Transl Med. 2019;7(Suppl 2):S64. doi: 10.21037/atm.2019.02.43 31179301 PMC6531649

[12] Yu XE, Gao S, Yang RM, Han YZ. MR Imaging of the Brain in Neurologic Wilson Disease. AJNR Am J Neuroradiol. 2019;40(1):178–83. doi: 10.3174/ajnr.A5936 30635331 PMC7048587

[13] Dong J, Wang X, Xu C, Gao M, Wang S, Zhang J, et al. Inhibiting NLRP3 inflammasome activation prevents copper-induced neuropathology in a murine model of Wilson’s disease. Cell Death Dis. 2021;12(1):87. doi: 10.1038/s41419-021-03397-1 33462188 PMC7813851

[14] Zimbrean PC, Schilsky ML. Psychiatric aspects of Wilson disease: a review. Gen Hosp Psychiatry. 2014;36(1):53–62. doi: 10.1016/j.genhosppsych.2013.08.007 24120023

[15] Guerrero-Jimenez M, Carrillo de Albornoz Calahorro CM, Gutierrez Rojas L. Wilson disease and psychiatric symptoms: A brief case report. Gen Psychiatr. 2019;32(3):e100066. doi: 10.1136/gpsych-2019-100066 31423476 PMC6677933

[16] Benhamla T, Tirouche YD, Abaoub-Germain A, Theodore F. [The onset of psychiatric disorders and Wilson’s disease]. Encephale. 2007;33(6):924–32. 18789784 10.1016/j.encep.2006.08.009

[17] Fu X, Zhang Y, Jiang W, Monnot AD, Bates CA, Zheng W. Regulation of copper transport crossing brain barrier systems by Cu-ATPases: effect of manganese exposure. Toxicol Sci. 2014;139(2):432–51. doi: 10.1093/toxsci/kfu048 24614235 PMC4064014

[18] Schmidt K, Ralle M, Schaffer T, Jayakanthan S, Bari B, Muchenditsi A, et al. ATP7A and ATP7B copper transporters have distinct functions in the regulation of neuronal dopamine-beta-hydroxylase. J Biol Chem. 2018;293(52):20085–98. 30341172 10.1074/jbc.RA118.004889 PMC6311498

[19] Barnes N, Tsivkovskii R, Tsivkovskaia N, Lutsenko S. The copper-transporting ATPases, menkes and wilson disease proteins, have distinct roles in adult and developing cerebellum. J Biol Chem. 2005;280(10):9640–5. doi: 10.1074/jbc.M413840200 15634671

[20] Dynamic Lutsenko S. and cell-specific transport networks for intracellular copper ions. J Cell Sci. 2021;134(21). 10.1242/jcs.240523 PMC8627558 34734631

[21] Huster D, Finegold MJ, Morgan CT, Burkhead JL, Nixon R, Vanderwerf SM, et al. Consequences of copper accumulation in the livers of the Atp7b-/- (Wilson disease gene) knockout mice. Am J Pathol. 2006;168(2):423–34. doi: 10.2353/ajpath.2006.050312 16436657 PMC1606493

[22] Buiakova OI, Xu J, Lutsenko S, Zeitlin S, Das K, Das S, et al. Null mutation of the murine ATP7B (Wilson disease) gene results in intracellular copper accumulation and late-onset hepatic nodular transformation. Hum Mol Genet. 1999;8(9):1665–71. doi: 10.1093/hmg/8.9.1665 10441329

[23] Allen KJ, Buck NE, Cheah DM, Gazeas S, Bhathal P, Mercer JF. Chronological changes in tissue copper, zinc and iron in the toxic milk mouse and effects of copper loading. Biometals. 2006;19(5):555–64. doi: 10.1007/s10534-005-5918-5 16937262

[24] Xie F, Xi Y, Pascual JM, Muzik O, Peng F. Age-dependent changes of cerebral copper metabolism in Atp7b (-/-) knockout mouse model of Wilson’s disease by [(64)Cu]CuCl(2)-PET/CT. Metab Brain Dis. 2017;32(3):717–26. doi: 10.1007/s11011-017-9956-9 28130615 PMC5573586

[25] Litwin T, Dusek P, Szafranski T, Dziezyc K, Czlonkowska A, Rybakowski JK. Psychiatric manifestations in Wilson’s disease: possibilities and difficulties for treatment. Ther Adv Psychopharmacol. 2018;8(7):199–211. doi: 10.1177/2045125318759461 29977520 PMC6022881

[26] Zheng G, Chen J, Zheng W. Relative contribution of CTR1 and DMT1 in copper transport by the blood-CSF barrier: implication in manganese-induced neurotoxicity. Toxicol Appl Pharmacol. 2012;260(3):285–93. doi: 10.1016/j.taap.2012.03.006 22465424 PMC3336026

[27] Rizzolo LJ. Polarization of the Na+, K(+)-ATPase in epithelia derived from the neuroepithelium. Int Rev Cytol. 1999;185:195–235. doi: 10.1016/s0074-7696(08)60152-7 9750268

[28] Gregoriades JMC, Madaris A, Alvarez FJ, Alvarez-Leefmans FJ. Genetic and pharmacological inactivation of apical Na(+)-K(+)-2Cl(-) cotransporter 1 in choroid plexus epithelial cells reveals the physiological function of the cotransporter. Am J Physiol Cell Physiol. 2019;316(4):C525–C44. doi: 10.1152/ajpcell.00026.2018 30576237 PMC6482671

[29] Donsante A, Johnson P, Jansen LA, Kaler SG. Somatic mosaicism in Menkes disease suggests choroid plexus-mediated copper transport to the developing brain. Am J Med Genet A. 2010;152A(10):2529–34. doi: 10.1002/ajmg.a.33632 20799318 PMC3117432

[30] Leary SC, Ralle M. Advances in visualization of copper in mammalian systems using X-ray fluorescence microscopy. Curr Opin Chem Biol. 2020;55:19–25. doi: 10.1016/j.cbpa.2019.12.002 31911338 PMC7237281

[31] Haddad MR, Choi EY, Zerfas PM, Yi L, Martinelli D, Sullivan P, et al. Cerebrospinal Fluid-Directed rAAV9-rsATP7A Plus Subcutaneous Copper Histidinate Advance Survival and Outcomes in a Menkes Disease Mouse Model. Mol Ther Methods Clin Dev. 2018;10:165–78. doi: 10.1016/j.omtm.2018.07.002 30090842 PMC6080355

[32] Nambiar R, McConnell RE, Tyska MJ. Myosin motor function: the ins and outs of actin-based membrane protrusions. Cell Mol Life Sci. 2010;67(8):1239–54. doi: 10.1007/s00018-009-0254-5 20107861 PMC3095969

[33] Heimsath EG Jr., Yim YI, Mustapha M, Hammer JA, Cheney RE. Myosin-X knockout is semi-lethal and demonstrates that myosin-X functions in neural tube closure, pigmentation, hyaloid vasculature regression, and filopodia formation. Sci Rep. 2017;7(1):17354. doi: 10.1038/s41598-017-17638-x 29229982 PMC5725431

[34] Stewart LC, Klinman JP. Dopamine beta-hydroxylase of adrenal chromaffin granules: structure and function. Annu Rev Biochem. 1988;57:551–92. doi: 10.1146/annurev.bi.57.070188.003003 3052283

[35] Ohno M, Narita T, Abe J, Tsuzuki T, Yagi K, Takikita S, et al. Apoptosis in cerebrum of macular mutant mouse. Acta Neuropathol. 2002;103(4):356–62. doi: 10.1007/s00401-001-0473-9 11904755

[36] Zecevic N, Verney C. Development of the catecholamine neurons in human embryos and fetuses, with special emphasis on the innervation of the cerebral cortex. J Comp Neurol. 1995;351(4):509–35. doi: 10.1002/cne.903510404 7721981

[37] Xiao T, Ackerman CM, Carroll EC, Jia S, Hoagland A, Chan J, et al. Copper regulates rest-activity cycles through the locus coeruleus-norepinephrine system. Nat Chem Biol. 2018;14(7):655–63. doi: 10.1038/s41589-018-0062-z 29867144 PMC6008210

[38] Horibata Y, Ando H, Sugimoto H. Locations and contributions of the phosphotransferases EPT1 and CEPT1 to the biosynthesis of ethanolamine phospholipids. J Lipid Res. 2020;61(8):1221–31. doi: 10.1194/jlr.RA120000898 32576654 PMC7397746

[39] Heissat S, Harel A, Um K, Brunet AS, Hervieu V, Guillaud O, et al. Evaluation of the accuracy of exchangeable copper and relative exchangeable copper (REC) in a mouse model of Wilson’s disease. J Trace Elem Med Biol. 2018;50:652–7. doi: 10.1016/j.jtemb.2018.06.013 30269758

[40] Batzios S, Tal G, DiStasio AT, Peng Y, Charalambous C, Nicolaides P, et al. Newly identified disorder of copper metabolism caused by variants in CTR1, a high-affinity copper transporter. Hum Mol Genet. 2022. doi: 10.1093/hmg/ddac156 35913762 PMC9759326

[41] Ralle M, Huster D, Vogt S, Schirrmeister W, Burkhead JL, Capps TR, et al. Wilson disease at a single cell level: intracellular copper trafficking activates compartment-specific responses in hepatocytes. J Biol Chem. 2010;285(40):30875–83. doi: 10.1074/jbc.M110.114447 20647314 PMC2945580

[42] Gray LW, Peng F, Molloy SA, Pendyala VS, Muchenditsi A, Muzik O, et al. Urinary copper elevation in a mouse model of Wilson’s disease is a regulated process to specifically decrease the hepatic copper load. PLoS One. 2012;7(6):e38327. doi: 10.1371/journal.pone.0038327 22802922 PMC3390108

[43] Pierson H, Muchenditsi A, Kim BE, Ralle M, Zachos N, Huster D, et al. The Function of ATPase Copper Transporter ATP7 B in Intestine. Gastroenterology. 2018;154(1):168–80 e5. 28958857 10.1053/j.gastro.2017.09.019 PMC5848507

[44] Ziemssen T, Akgun K, Czlonkowska A, Antos A, Bembenek J, Kurkowska-Jastrzebska I, et al. Serum Neurofilament Light Chain as a Biomarker of Brain Injury in Wilson’s Disease: Clinical and Neuroradiological Correlations. Mov Disord. 2022;37(5):1074–9. doi: 10.1002/mds.28946 35114010

[45] Kumar D, Strenkert D, Patel-King RS, Leonard MT, Merchant SS, Mains RE, et al. A bioactive peptide amidating enzyme is required for ciliogenesis. Elife. 2017;6. doi: 10.7554/eLife.25728 28513435 PMC5461114

[46] Rhodes CH, Xu RY, Angeletti RH. Peptidylglycine alpha-amidating monooxygenase (PAM) in Schwann cells and glia as well as neurons. J Histochem Cytochem. 1990;38(9):1301–11. doi: 10.1177/38.9.2387985 2387985

[47] Luxmi R, Kumar D, Mains RE, King SM, Eipper BA. Cilia-based peptidergic signaling. PLoS Biol. 2019;17(12):e3000566. doi: 10.1371/journal.pbio.3000566 31809498 PMC6919629

[48] Fujiwara N, Iso H, Kitanaka N, Kitanaka J, Eguchi H, Ookawara T, et al. Effects of copper metabolism on neurological functions in Wistar and Wilson’s disease model rats. Biochem Biophys Res Commun. 2006;349(3):1079–86. doi: 10.1016/j.bbrc.2006.08.139 16970921

[49] Saito T, Nagao T, Okabe M, Saito K. Neurochemical and histochemical evidence for an abnormal catecholamine metabolism in the cerebral cortex of the Long-Evans Cinnamon rat before excessive copper accumulation in the brain. Neurosci Lett. 1996;216(3):195–8. doi: 10.1016/0304-3940(96)13041-x 8897491

[50] Nyberg P, Gottfries CG, Holmgren G, Persson S, Roos BE, Winblad B. Advanced catecholaminergic disturbances in the brain in a case of Wilson’s disease. Acta Neurol Scand. 1982;65(1):71–5. doi: 10.1111/j.1600-0404.1982.tb03063.x 6175159

[51] Mazi TA, Shibata NM, Medici V. Lipid and energy metabolism in Wilson disease. Liver Res. 2020;4(1):5–14. doi: 10.1016/j.livres.2020.02.002 32832193 PMC7437987

[52] Huster D, Lutsenko S. Wilson disease: not just a copper disorder. Analysis of a Wilson disease model demonstrates the link between copper and lipid metabolism. Mol Biosyst. 2007;3(12):816–24. doi: 10.1039/b711118p 18000558

[53] Guttmann S, Nadzemova O, Grunewald I, Lenders M, Brand E, Zibert A, et al. ATP7B knockout disturbs copper and lipid metabolism in Caco-2 cells. PLoS One. 2020;15(3):e0230025. doi: 10.1371/journal.pone.0230025 32155648 PMC7064347

[54] Krishnamoorthy L, Cotruvo JA Jr., Chan J, Kaluarachchi H, Muchenditsi A, Pendyala VS, et al. Copper regulates cyclic-AMP-dependent lipolysis. Nat Chem Biol. 2016;12(8):586–92. doi: 10.1038/nchembio.2098 27272565 PMC4955676

[55] Hara A, Taketomi T. Cerebral lipid and protein abnormalities in Menkes’ steely-hair disease. Jpn J Exp Med. 1986;56(6):277–84. 3599492

[56] Mazi TA, Sarode GV, Czlonkowska A, Litwin T, Kim K, Shibata NM, et al. Dysregulated Choline, Methionine, and Aromatic Amino Acid Metabolism in Patients with Wilson Disease: Exploratory Metabolomic Profiling and Implications for Hepatic and Neurologic Phenotypes. Int J Mol Sci. 2019;20(23). doi: 10.3390/ijms20235937 31779102 PMC6928853

[57] Medici V, Shibata NM, Kharbanda KK, Islam MS, Keen CL, Kim K, et al. Maternal choline modifies fetal liver copper, gene expression, DNA methylation, and neonatal growth in the tx-j mouse model of Wilson disease. Epigenetics. 2014;9(2):286–96. doi: 10.4161/epi.27110 24220304 PMC3962539

[58] Semple BD, Blomgren K, Gimlin K, Ferriero DM, Noble-Haeusslein LJ. Brain development in rodents and humans: Identifying benchmarks of maturation and vulnerability to injury across species. Prog Neurobiol. 2013;106–107:1–16. doi: 10.1016/j.pneurobio.2013.04.001 23583307 PMC3737272

[59] Muchenditsi A, Talbot CC Jr., Gottlieb A, Yang H, Kang B, Boronina T, et al. Systemic deletion of Atp7b modifies the hepatocytes’ response to copper overload in the mouse models of Wilson disease. Sci Rep. 2021;11(1):5659. doi: 10.1038/s41598-021-84894-3 33707579 PMC7952580

[60] Shariatgorji M, Nilsson A, Kallback P, Karlsson O, Zhang X, Svenningsson P, et al. Pyrylium Salts as Reactive Matrices for MALDI-MS Imaging of Biologically Active Primary Amines. J Am Soc Mass Spectrom. 2015;26(6):934–9. doi: 10.1007/s13361-015-1119-9 25821050

[61] Seneviratne HK, Hendrix CW, Fuchs EJ, Bumpus NN. MALDI Mass Spectrometry Imaging Reveals Heterogeneous Distribution of Tenofovir and Tenofovir Diphosphate in Colorectal Tissue of Subjects Receiving a Tenofovir-Containing Enema. J Pharmacol Exp Ther. 2018;367(1):40–8. doi: 10.1124/jpet.118.250357 30037813 PMC6123665

[62] Karuppagounder SS, Brahmachari S, Lee Y, Dawson VL, Dawson TM, Ko HS. The c-Abl inhibitor, nilotinib, protects dopaminergic neurons in a preclinical animal model of Parkinson’s disease. Sci Rep. 2014;4:4874. doi: 10.1038/srep04874 24786396 PMC4007078

[63] Jelinek J, Jensen A. Catecholamine concentrations in plasma and organs of the fetal guinea pig during normoxemia, hypoxemia, and asphyxia. J Dev Physiol. 1991;15(3):145–52. 1940141

[64] Matyash V, Liebisch G, Kurzchalia TV, Shevchenko A, Schwudke D. Lipid extraction by methyl-tert-butyl ether for high-throughput lipidomics. J Lipid Res. 2008;49(5):1137–46. doi: 10.1194/jlr.D700041-JLR200 18281723 PMC2311442

[65] Chong J, Wishart DS, Xia J. Using MetaboAnalyst 4.0 for Comprehensive and Integrative Metabolomics Data Analysis. Curr Protoc Bioinformatics. 2019;68(1):e86. doi: 10.1002/cpbi.86 31756036

[66] Xia J, Wishart DS. Metabolomic data processing, analysis, and interpretation using MetaboAnalyst. Curr Protoc Bioinformatics. 2011;Chapter 14:Unit 14 0. doi: 10.1002/0471250953.bi1410s34 21633943

