# Supplementary material for: Atp7b-dependent choroid plexus dysfunction causes transient copper deficit and metabolic changes in the developing mouse brain
Source: PLoS Genet. 2023 Jan 10;19(1):e1010558. doi: 10.1371/journal.pgen.1010558 (PMC9870141; doi:10.1371/journal.pgen.1010558)
Supplement: S1 Fig — Localization of Ctr1 at the apical membrane and the basolateral (stromal) aspect of ChPl (a) Immunofluorescent staining for Ctr1 (Red) and apical membrane marker NKCC1 (Green) on ChPl from 4 weeks Atp7b-/- and Wild type. ChPl was co-stained with F-actin (Phalloidin_Alex647; magenta). The Ctr1 shows colocalization with NKCC1 in wild type ChPl on the apical membrane which was completely absent in Atp7b-/-. Ctr1 staining was also observed on the basolateral membrane of ChPl. The magnified area was marked with a white dotted box. The apical membrane has been marked with white arrows. (b) The colocalization of Ctr1 with NKCC1 and F actin was shown using RGB profile plot. The area for which the RGB profile plot was generated has been shown with a white line. Scale bar 20 μm. (PDF) [file pgen.1010558.s001.pdf]

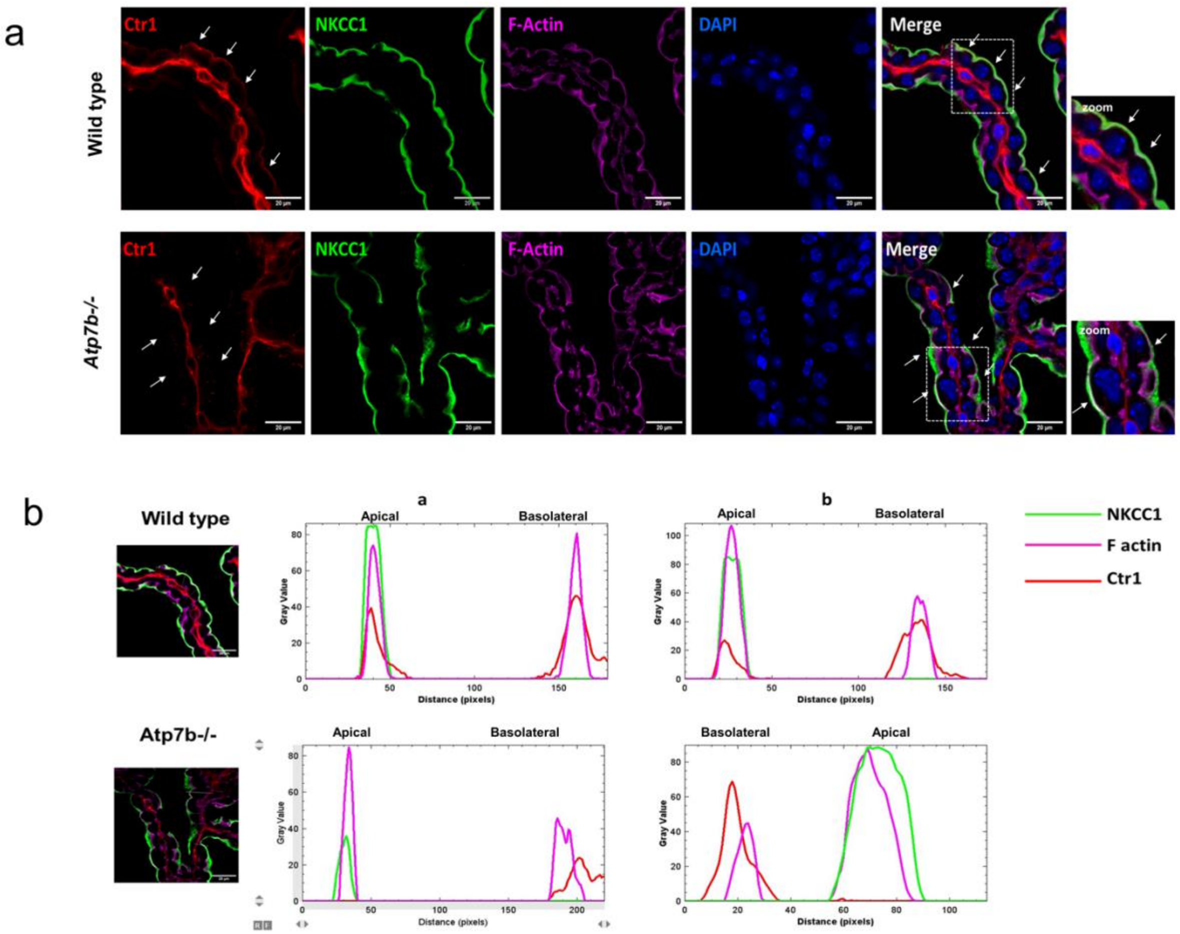

S1_Fig. Localization of Ctr1 at the apical membrane and the basolateral (stromal) aspect of ChPl (a) Immunofluorescent staining for Ctr1 (Red) and apical membrane marker NKCC1 (Green) on ChPl from 4 weeks Atp7b-/- and Wild type. ChPl was co-stained with F-actin (Phalloidin_Alex647; magenta). The Ctr1 shows colocalization with NKCC1 in wild type ChPl on the apical membrane which was completely absent in Atp7b-/-. Ctr1 staining was also observed on the basolateral membrane of ChPl. The magnified area was marked with a white dotted box. The apical membrane has been marked with white arrows. (b) The colocalization of Ctr1 with NKCC1 and F actin was shown using RGB profile plot. The area for which the RGB profile plot was generated has been shown with a white line. Scale bar 20 μm.
