# Supplementary material for: Atp7b-dependent choroid plexus dysfunction causes transient copper deficit and metabolic changes in the developing mouse brain
Source: PLoS Genet. 2023 Jan 10;19(1):e1010558. doi: 10.1371/journal.pgen.1010558 (PMC9870141; doi:10.1371/journal.pgen.1010558)
Supplement: S2 Fig — Cilia density is decreased in the 4 week-old Atp7b-/- ChPl compared to controls (a) Immunofluorescent staining for ciliary membrane marker Arl13b (green), gamma tubulin (red) on ChPl from 4 weeks Atp7b-/- and Wild type controls. The data here for Arl13b/γ-tub/DAPI merged is the same as in Fig 3G. Scale Bar 20 μm (b) Merged Z stack images for Arl13b (white) and gamma tubulin (red) from Wild type and Atp7b-/- ChPl were used to count the number of ciliated cells. The ciliated structures were marked using a white box. Scale Bar 20 μm (c) Percentage of ciliated epithelial cells between Atp7b-/- and wild type ChPl were calculated from these images. Data represented as Means ± SD. *P < 0.001 (Welch corrected t-test). n = 3–4 (PDF) [file pgen.1010558.s002.pdf]

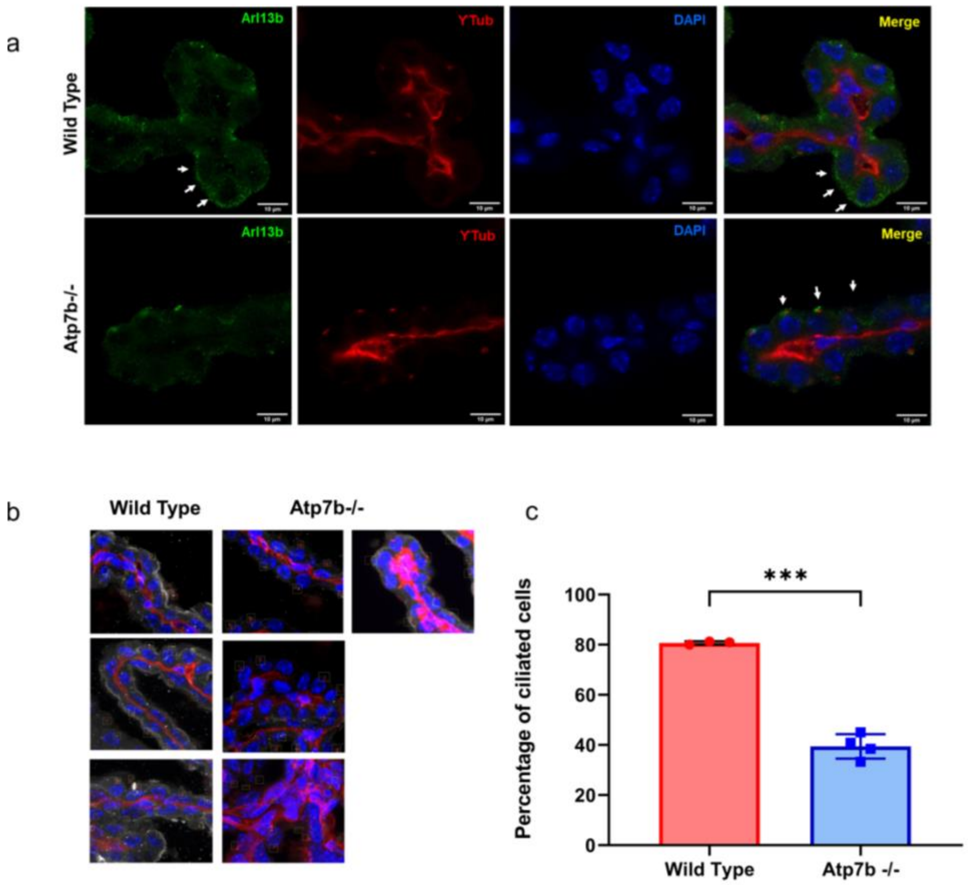

S2_Fig. Cilia density is decreased in the 4 week-old *Atp7b*$^{-/-}$ ChPl compared to controls
(a) Immunofluorescent staining for ciliary membrane marker Arl13b (green), gamma tubulin (red) on ChPl from 4 weeks Atp7b-/- and Wild type controls. The data here for Arl13b/ϒtub/DAPI merged is the same as in Figure 3g. Scale Bar 20 μm **(b)** Merged Z stack images for Arl13b (white) and gamma tubulin (red) from Wild type and Atp7b-/- ChPl were used to count the number of ciliated cells. The ciliated structures were marked using a white box. Scale Bar 20 μm **(c)** Percentage of ciliated epithelial cells between Atp7b-/- and wild type ChPl were calculated from these images. Data represented as Means ± SD. $*P < 0.001$ (Welch corrected *t*-test). n = 3-4
