# Supplementary material for: Atp7b-dependent choroid plexus dysfunction causes transient copper deficit and metabolic changes in the developing mouse brain
Source: PLoS Genet. 2023 Jan 10;19(1):e1010558. doi: 10.1371/journal.pgen.1010558 (PMC9870141; doi:10.1371/journal.pgen.1010558)
Supplement: S3 Fig — Immunofluorescent staining for focal adhesion kinase (FAK) on ChPl from 4 weeks Atp7b-/- and Wild type controls. The ChPl was stained for FAK (green), Transthyretin (TTR) (Red), and F-actin (Magenta). The data here for FAK is the same as in Fig 4C. The quantitation for FAK shown in Fig 4D was performed by normalizing the fluorescent intensity of FAK to F-actin. Scale bar 20 μm. S4 Fig. Quantification of DBH-positive cell located at both sides of the fourth ventricle in 4-weeks-old control C57Bl/6 mice. (A) Representative cross sections and DBH staining at various distances from a complete closing of the fourth ventricle. (B) DBH-positive cells on both sides of the fourth ventricle were quantified and plotted using individuate sample replicates. (C) Average of DBH-positive cells at various distances from the complete closing of the fourth ventricle. (PDF) [file pgen.1010558.s003.pdf]

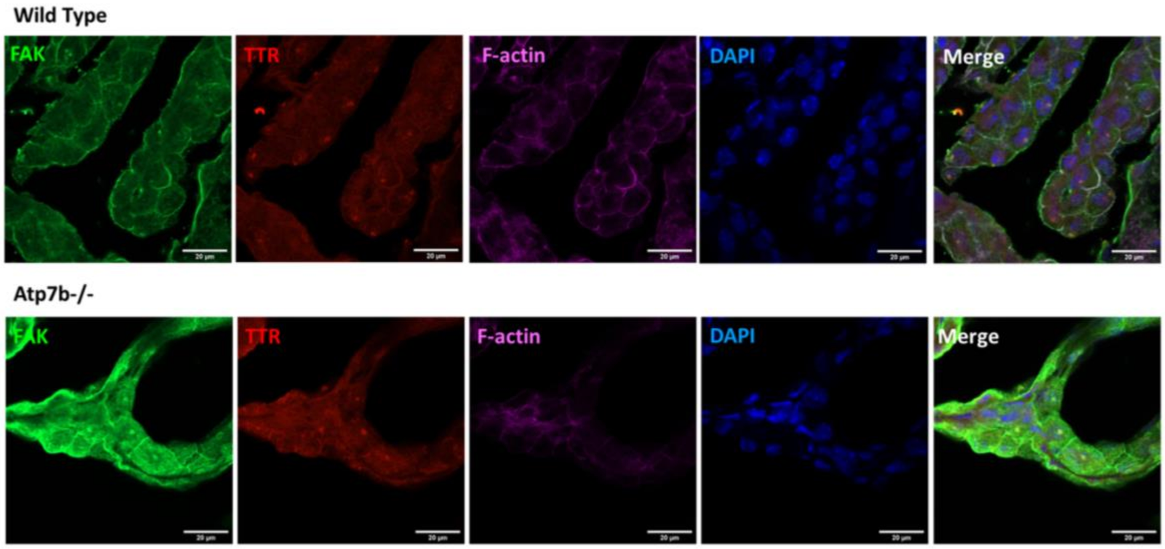

S3_Figure (extended Fig.4c). Immunofluorescent staining for focal adhesion kinase (FAK) on ChPl from 4 weeks *Atp7b-/-* and Wild type controls. The ChPl was stained for FAK (green), Transthyretin (TTR) (Red), and F-actin (Magenta). The data here for FAK is the same as in Figure 4c. The quantitation for FAK shown in Figure 4d was performed by normalizing the fluorescent intensity of FAK to F-actin. Scale bar 20 μm.
