# Supplementary material for: Atp7b-dependent choroid plexus dysfunction causes transient copper deficit and metabolic changes in the developing mouse brain
Source: PLoS Genet. 2023 Jan 10;19(1):e1010558. doi: 10.1371/journal.pgen.1010558 (PMC9870141; doi:10.1371/journal.pgen.1010558)
Supplement: S4 Fig — (A) Representative cross sections and DBH staining at various distances from a complete closing of the fourth ventricle. (B) DBH-positive cells on both sides of the fourth ventricle were quantified and plotted using individuate sample replicates. (C) Average of DBH-positive cells at various distances from the complete closing of the fourth ventricle. (PDF) [file pgen.1010558.s004.pdf]

SUPPLEMENTAL FIGURES

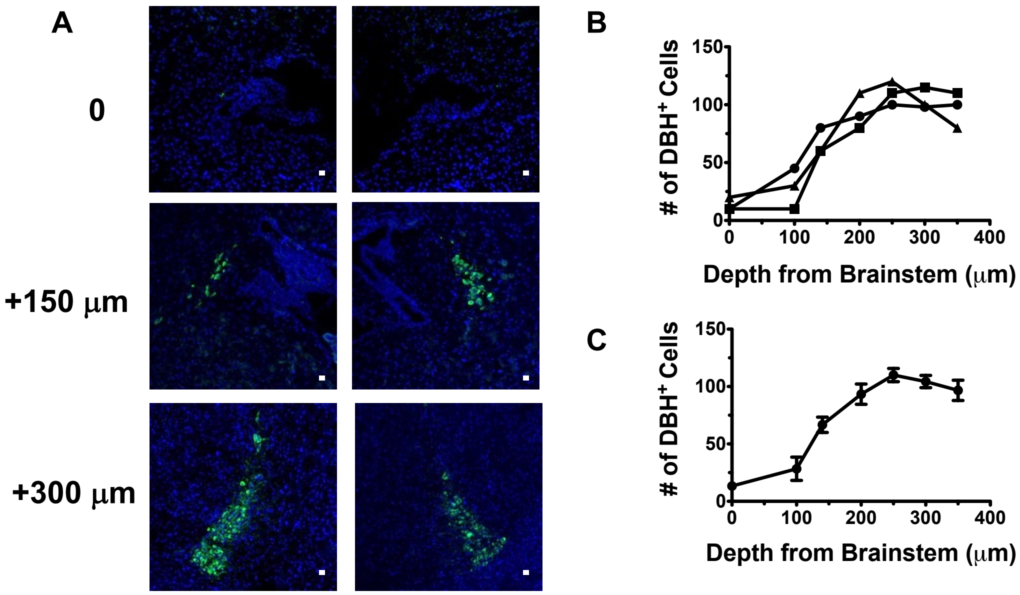

S4 Fig. Quantification of DBH-positive cell located at both sides of the fourth ventricle in 4-weeks-old control C57Bl/6 mice. (A) Representative cross sections and DBH staining at various distances from a complete closing of the fourth ventricle. **(B)** DBH-positive cells on both sides of the fourth ventricle were quantified and plotted using individuate sample replicates. **(C)** Average of DBH-positive cells at various distances from the complete closing of the fourth ventricle.
