# Supplementary material for: Atp7b-dependent choroid plexus dysfunction causes transient copper deficit and metabolic changes in the developing mouse brain
Source: PLoS Genet. 2023 Jan 10;19(1):e1010558. doi: 10.1371/journal.pgen.1010558 (PMC9870141; doi:10.1371/journal.pgen.1010558)
Supplement: S5 Fig — The data for Atp7a at 4 weeks here is the same as in the Fig 5B. Scale bar = 20μm. (PDF) [file pgen.1010558.s005.pdf]

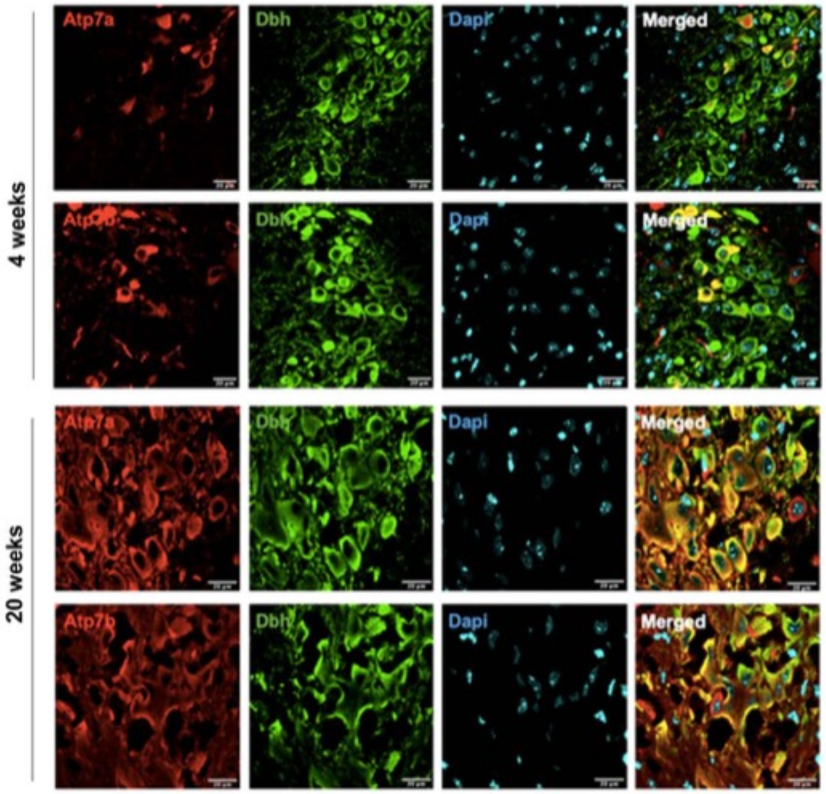

S5 Fig. Expression of Atp7a and Atp7b (red) in DBH positive cells (green) of locus coeruleus at 4 weeks and 20 weeks after birth. The data for Atp7a at 4 weeks here is the same as in the Fig.5b. Scale bar = 20mm
