# Supplementary material for: Atp7b-dependent choroid plexus dysfunction causes transient copper deficit and metabolic changes in the developing mouse brain
Source: PLoS Genet. 2023 Jan 10;19(1):e1010558. doi: 10.1371/journal.pgen.1010558 (PMC9870141; doi:10.1371/journal.pgen.1010558)
Supplement: S6 Fig — CHCA was used as the matrix. (bottom panels) In order to confirm the identities of derivatized DA and NE, collision-induced dissociation was carried out. From these experiments, derivatized DA exhibited fragment ions at m/z 122.0958 and 137.0591 corresponding to C8H12N+ and C8H8O2+, respectively. Similarly, derivatized NE yielded its fragment ions at m/z 122.0959 and 153.0540 corresponding to C8H12N+ and C8H8O3+, respectively. (PDF) [file pgen.1010558.s006.pdf]

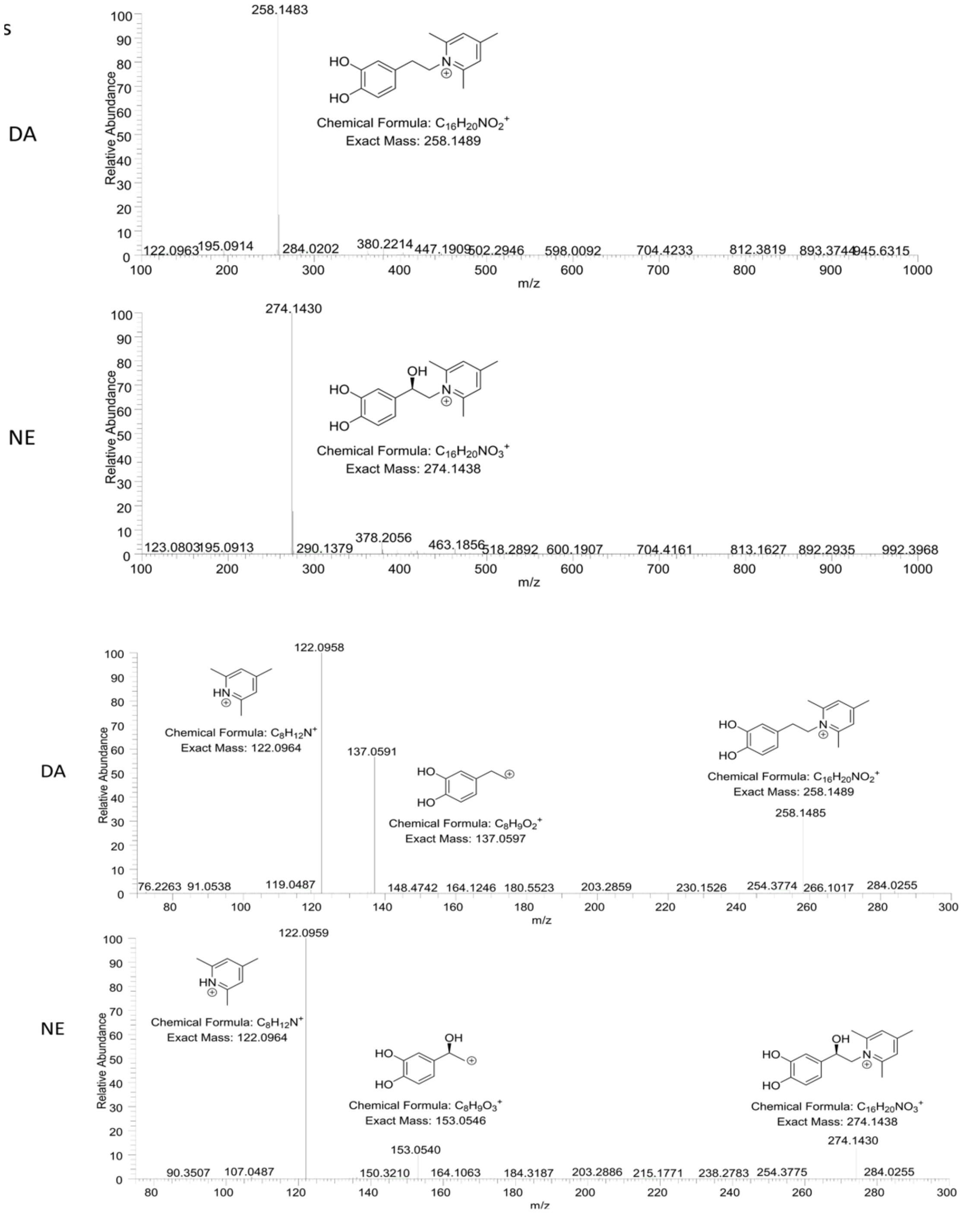

S6 Fig. (top two panels) Representative average full scan mass spectra of derivatized DA and NE in brain tissue exhibiting the detection of their ions at *m/z* 258.1485 and 274.1435, respectively. CHCA was used as the matrix. (bottom panels) In order to confirm the identities of derivatized DA and NE, collision-induced dissociation was carried out. From these experiments, derivatized DA exhibited fragment ions at *m/z* 122.0958 and 137.0591 corresponding to $C_8H_{12}N^+$ and $C_8H_8O_2^+$, respectively. Similarly, derivatized NE yielded its fragment ions at m/z 122.0959 and 153.0540 corresponding to $C_8H_{12}N^+$ and $C_8H_8O_3^+$, respectively.
