# Supplementary material for: Atp7b-dependent choroid plexus dysfunction causes transient copper deficit and metabolic changes in the developing mouse brain
Source: PLoS Genet. 2023 Jan 10;19(1):e1010558. doi: 10.1371/journal.pgen.1010558 (PMC9870141; doi:10.1371/journal.pgen.1010558)
Supplement: S7 Fig — 5-HIAA is 5-hydroxyindoleacetic acid, DOPAC—3,4-dihydroxyphenylacetic acid, 5HT—5-hydroxytryptamine, 3MT—3-homovanillic acid (n = 3–4) (PDF) [file pgen.1010558.s007.pdf]

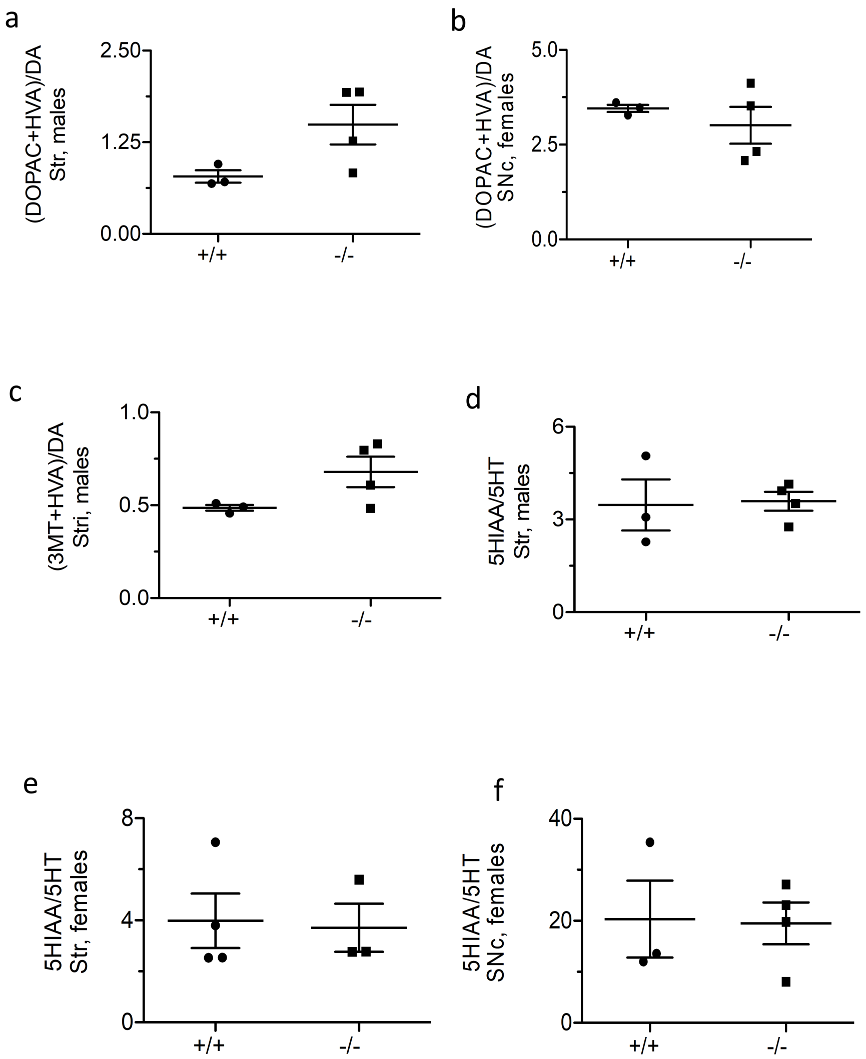

S7 Fig. Analysis of dopamine degradation products in striatum (Str) and substantia nigra (SNc) in males and females. 5-HIAA is 5-hydroxyindoleacetic acid, DOPAC - 3,4-dihydroxyphenylacetic acid, 5HT - 5-hydroxytryptamine, 3MT - 3-homovanillic acid (n=3-4)
