# Supplementary material for: Atp7b-dependent choroid plexus dysfunction causes transient copper deficit and metabolic changes in the developing mouse brain
Source: PLoS Genet. 2023 Jan 10;19(1):e1010558. doi: 10.1371/journal.pgen.1010558 (PMC9870141; doi:10.1371/journal.pgen.1010558)
Supplement: S1 Text — (PDF) [file pgen.1010558.s008.pdf]

Supplement

Atp7b-Dependent Choroid Plexus Dysfunction Causes Transient Copper Deficit and Metabolic Changes in the Developing Mouse Brain

Clorissa L. Washington-Hughes[1], Shubhrajit Roy[1], Herana K. Seneviratne[2], Senthilkumar S. Karuppagounder[3,4], Yulemni Morel[5], Jace W. Jones[5], Alex Zak[1], Tong Xiao[6], Tatiana Boronina, Robert Cole, Namandjé N. Bumpus[2], Chris Chang[6], Ted M. Dawson[3,4], and Svetlana Lutsenko[1#]

Supplemental Methods

Lipid analysis

Materials: LC-MS grade acetonitrile, methanol, water, isopropanol, ammonium acetate, and ammonium fluoride were purchased from Fisher Scientific (Pittsburg, PA). HPLC grade tert-Butyl methyl ether (MTBE) was purchased from Sigma Aldrich (St. Louis, MO). EquiSPLASH lipidomix was purchased from Avanti Polar Lipids, Inc. (Alabaster, AL).

The LC-HRMS analyses were performed on an Agilent 1290 Infinity LC coupled to an Agilent 6560 Quadrupole Time-of-Flight (Q-TOF) mass spectrometer. The separation was achieved using an C18 CSH (1.7 µm; 2.1 x 100 mm) column (Waters, Milford, MA). Mobile phase A was 10 mM ammonium formate with 0.1% formic acid in water/acetonitrile (40:60, v/v) and mobile phase B was 10 mM ammonium formate with 0.1% formic acid in acetonitrile/isopropanol (10:90, v/v). The gradient was ramped from 40 % to 43 % B in 1 min, ramped to 50 % in 0.1 min, ramped to 54 % B in 4.9 minutes, ramped to 70 % in 0.1 min, and ramped to 99 % B in 2.9 min. The gradient was returned to initial conditions in 0.5 min and held for 1.6 min for column equilibration. The flow rate was 0.4 mL/min. The column was maintained at 55 °C and the auto-sampler was kept at 5 °C. A 2 µL injection was used for all samples.

Mass Spectrometry Analysis of ChPl proteome

Buffer exchange: Samples in 12.5uL 8M Urea, TEAB/0.75%SDS, BME, were precipitated with 8 volumes of TCA acetone at -20C overnight, washed with 2 volume of acetone. Proteins were resuspended in 18uL 75mM TEAB / 10%acetonitile and proteolyzed at 37C overnight with 400ng trypsin (Pierce). TMT labeling: Peptides from each sample were labeled with TMT10plex reagents according to manufacturer's instruction. Briefly 8.5 ul of TMT labels resuspended in 40 ul acetonitrile (anhydrous) were added to tryptic peptides in 20uL 100mM TEAB. After 1 hr, labeling was quenched with 1.6uL 5% hydroxylamine for 15min. Labeled samples were combined, dried, and loaded in 25uL 50mM TEAB onto Pierce detergent removal spin column (#87776, ThermoPierce) to remove excess TMT reagents and other potential small molecules or hydrophobic contaminants. Peptides were eluted in 25uL TEAB, acidified, desalted on u-HLB oasis plate in 150uL 0.1%TFA, eluted in 60% acetonitrile/0.1%TFA and dried.

Mass Spectrometry: The combined eight TMT labeled peptide sample were resuspended in 2% acetonitrile in 0.1% formic acid and analysed by liquid chromatography interfaced with tandem mass spectrometry (LCMSMS) using a EasyLC 1200 HPLC system (www.thermofisher.com) interfaced with a Orbitrap Fusion Lumos-ETD (www.thermofisher.com). Peptides were loaded onto a C18 trap (S-10µM, 120Å, 75 µm x 2 cm, YMC, Japan) for 5 min at 5ml/min in 2%

acetonitrile/0.1% formic acid in-line with a 75 um x 150 mm ProntoSIL-120-5-C18 H column (5µm, 120Å (BISCHOFF), www.bischoff-chrom.com). Peptides eluting during the 2%-90% acetonitrile in 0.1% formic acid gradient over 105 min at 300nl/min were directly sprayed into a Lumos-ETD mass spectrometer through 1 µm emitter tip (New Objective, www.newobjective.com) at 2.5 kV. Survey scans (full ms) were acquired from 375-1600 m/z with data dependent monitoring of up to 15 peptide masses (precursor ions), each individually isolated in a 0.7 Da window and fragmented using HCD activation collision energy at 38 and 15 s dynamic exclusion.

Precursor and fragment ions were analyzed at resolutions 120,000 and 60,000, respectively, MSMS data processing isotopically resolved masses in precursor (MS) and fragmentation (MS/MS) spectra were extracted from raw MS data in Proteome Discoverer (PD) software (v2.4, ThermoFisher Scientific) and searched, using Mascot v.2.8.1 (www.matrixscience.com) against the RefSeq2021_204_mus musculus and NCBI_210512 protein databases with proteolytic enzymes and BSA added. Search criteria included: all species; trypsin as enzyme, a maximum of two missed cleavage; TMT on N-terminus and lysine as fixed modifications; DeStreak on cysteine, methionine oxidation, asparagine and glutamine deamidation as variable modifications.

Peptide identifications from Mascot searches were filtered at 1% False Discovery Rate (FDR), based on a concatenated decoy database search, using the Proteome Discoverer andPercolator validation. Only Unique peptides with Rank 1 were considered. Proteome Discoverer uses TMT reporter ions from each peptide matched spectrum (PSM). PSMs with reporter ion signal/noise >5 and precursor solation interference <25 were used for quantification. Protein quantification was based on the normalized median ratio of all spectra of tagged peptides from the same protein (1). Peptides modified with methionine oxidation, asparagine and glutamine deamidation were excluded from normalization calculations.

Supplemental references:

1. Herbrich, S. M., Cole, R. N., West, K. P., Jr., Schulze, K., Yager, J. D., Groopman, J. D., Christian, P., Wu, L., O'Meally, R. N., May, D. H., McIntosh, M. W., and Ruczinski, I. (2013) Statistical inference from multiple iTRAQ experiments without using common reference standards. *J Proteome Res* **12**, 594-604
