# Supplementary material for: Atp7b-dependent choroid plexus dysfunction causes transient copper deficit and metabolic changes in the developing mouse brain
Source: PLoS Genet. 2023 Jan 10;19(1):e1010558. doi: 10.1371/journal.pgen.1010558 (PMC9870141; doi:10.1371/journal.pgen.1010558)
Supplement: S1 Table — Peach color indicate proteins upregulated more than 1.25 fold; green color–proteins downregulated more than 1.25 fold. (PDF) [file pgen.1010558.s009.pdf]

Supplemental Table

Significant changes in protein abundances in *Atp7b*$^{-/-}$ ChPl compared to control (p-value<0.1)
Peach color indicate proteins upregulated more than 1.25 fold; green color – proteins downregulated more than 1.25 fold

Accession #	Cellular Functions	Fold Change	p-value
	**Mitochondria function**		
MRPL1	mitochondrial ribosomal protein L1	1.672	0.06141939
DLAT	dihydrolipoamide S-acetyltransferase	1.592	0.04102858
SOD1	superoxide dismutase 1	1.519	0.0375117
NDUFV2	NADH:ubiquinone oxidoreductase core subunit V2	1.477	0.07880414
HSPD1	heat shock protein family D (Hsp60) member 1	1.323	0.03634122
ATP5F1B	ATP synthase F1 subunit beta	1.294	0.06484241
	**RNA processing**		
PTBP1	polypyrimidine tract binding protein 1	1.399	0.03133123
SFPQ	splicing factor proline and glutamine rich	1.385	0.04187421
Srrm1	serine/arginine repetitive matrix 1	1.374	0.01336447
PRRC2C	proline rich coiled-coil 2C	1.331	0.08692178
HNRNPM	heterogeneous nuclear ribonucleoprotein M	1.316	0.01495869
EEF2	eukaryotic translation elongation factor 2	1.284	0.02204163
MATR3	matrin 3	1.278	0.06513348
EPRS1	glutamyl-prolyl-tRNA synthetase 1	1.273	0.07917095
Fip1l1	FIP1 like 1 (S. cerevisiae)	1.267	0.0426433
DARS1	aspartyl-tRNA synthetase 1	-1.727	0.00020146
	**Regulation of transcription**		
PBXIP1	PBX homeobox interacting protein 1	1.4	0.0706412
LMO7	LIM domain 7	1.393	0.056518
YLPM1	YLP motif containing 1	1.361	0.0624536
DHX9	DExH-box helicase 9	1.266	0.03645957
BRAF	B-Raf proto-oncogene, serine/threonine kinase	-1.339	0.09378855
DCTN2	dynactin subunit 2	-1.348	0.09098471
CENPV	centromere protein V	-1.433	0.03416556
	**Stress response, ion balance, osmolarity**		
Lta4h	leukotriene A-4 hydrolase	1.442	0.01597781
SLC12A5	solute carrier family 12 member 5	1.442	0.0243286
PRRC2C	proline rich coiled-coil 2C	1.331	0.08692178
ALDOA	aldolase, fructose-bisphosphate A	-1.342	0.07619066
HSP90AB1	heat shock protein 90 alpha family class B member 1	-1.577	0.00416622
ACE	angiotensin I converting enzyme	-1.764	0.07114454
ALB	albumin	-2.331	0.00406153
HBA1/HBA2	hemoglobin subunit alpha 2	-4.587	0.03085708
HBB	hemoglobin subunit beta	-6.135	0.00367993
	**Proteolysis**		
COPS3	COP9 signalosome subunit 3	1.368	0.0034167
PSMA1	proteasome 20S subunit alpha 1	1.366	0.05786659
ADRM1	adhesion regulating molecule 1	-1.412	0.04632558
SERPINA1	serpin family A member 1	-3.623	0.01953355
SERPINA3	serpin family A member 3	-1.562	0.06567966

Cytoskeleton, cell adhesion, cell-cell contacts			
DSG1	desmoglein 1	2.669	0.08847092
PTK2	protein tyrosine kinase 2	2.522	0.01993896
MYH11	myosin heavy chain 11	1.853	0.013013
KRT1	keratin 1	1.805	0.07364956
SYNGR1	synaptogyrin 1	1.766	0.07281514
VIM	vimentin	1.615	0.02798218
JUP	junction plakoglobin	1.603	0.02112259
LAMB2	laminin subunit beta 2	1.566	0.00522966
ENO1	enolase 1	1.518	0.07439016
HSPG2	heparan sulfate proteoglycan 2	1.514	0.03462512
TJP2	tight junction protein 2	1.428	0.04594531
PLVAP	plasmalemma vesicle associated protein	1.388	0.06449303
COL5A2	collagen type V alpha 2 chain	1.341	0.0188553
PLEC	plectin	1.338	0.01847884
MPP2	membrane palmitoylated protein 2	1.338	0.09525415
GSN	gelsolin	1.335	0.09417545
CNST	consortin, connexin sorting protein	1.325	0.00470009
TLN1	talin 1	1.316	0.05808765
Cdc42	cell division cycle 42	1.307	0.06140247
LAMC1	laminin subunit gamma 1	1.284	0.04296727
ACTN1	actinin alpha 1	1.275	0.09806463
KRT16	keratin 16	1.27	0.02822053
NEFH	neurofilament heavy	-1.344	0.063195
DCTN2	dynactin subunit 2	-1.348	0.09098471
SPTBN1	spectrin beta, non-erythrocytic 1	-1.377	0.06266399
ACTB	actin beta	-1.458	0.00838762
HOOK1	hook microtubule tethering protein 1	-1.513	0.02757636
EPPK1	epiplakin 1	-1.517	0.04594695
MYH9	myosin heavy chain 9	-1.522	0.03213158
GDI1	GDP dissociation inhibitor 1	-1.653	0.03798417
NEFL	neurofilament light	-1.684	0.07143479
C3	complement C3	-5.714	0.00373037
